# Pre-Steady-State Kinetics of the SARS-CoV-2 Main Protease as a Powerful Tool for Antiviral Drug Discovery

**DOI:** 10.3389/fphar.2021.773198

**Published:** 2021-12-06

**Authors:** Maria Yu. Zakharova, Alexandra A. Kuznetsova, Victoria I. Uvarova, Anastasiia D. Fomina, Liubov I. Kozlovskaya, Elena N. Kaliberda, Inna N. Kurbatskaia, Ivan V. Smirnov, Anatoly A. Bulygin, Vera D. Knorre, Olga S. Fedorova, Alexandre Varnek, Dmitry I. Osolodkin, Aydar A. Ishmukhametov, Alexey M. Egorov, Alexander G. Gabibov, Nikita A. Kuznetsov

**Affiliations:** ^1^ Institute of Bioorganic Chemistry, Russian Academy of Sciences (RAS), Moscow, Russia; ^2^ Institute of Translational Medicine, Pirogov Russian National Research Medical University, Moscow, Russia; ^3^ Institute of Chemical Biology and Fundamental Medicine, Siberian Branch (SB) of RAS, Novosibirsk, Russia; ^4^ FSASI “Chumakov FSC R&D IBP RAS” (Institute of Poliomyelitis), Moscow, Russia; ^5^ Lomonosov Moscow State University, Moscow, Russia; ^6^ Laboratoire de Chémoinformatique, UMR 7140 CNRS, Université de Strasbourg, Strasbourg, France; ^7^ Institute of Translational Medicine and Biotechnology, Sechenov First Moscow State Medical University, Moscow, Russia; ^8^ Department of Biology and Biotechnology, Higher School of Economics, Moscow, Russia; ^9^ Department of Natural Sciences, Novosibirsk State University, Novosibirsk, Russia

**Keywords:** SARS-CoV-2, main protease, pre-steady-state kinetics, substrate cleavage, inhibitor binding, molecular docking, stopped-flow

## Abstract

The design of effective target-specific drugs for COVID-19 treatment has become an intriguing challenge for modern science. The SARS-CoV-2 main protease, M^pro^, responsible for the processing of SARS-CoV-2 polyproteins and production of individual components of viral replication machinery, is an attractive candidate target for drug discovery. Specific M^pro^ inhibitors have turned out to be promising anticoronaviral agents. Thus, an effective platform for quantitative screening of M^pro^-targeting molecules is urgently needed. Here, we propose a pre–steady-state kinetic analysis of the interaction of M^pro^ with inhibitors as a basis for such a platform. We examined the kinetic mechanism of peptide substrate binding and cleavage by wild-type M^pro^ and by its catalytically inactive mutant C145A. The enzyme induces conformational changes of the peptide during the reaction. The inhibition of M^pro^ by boceprevir, telaprevir, GC-376, PF-00835231, or thimerosal was investigated. Detailed pre–steady-state kinetics of the interaction of the wild-type enzyme with the most potent inhibitor, PF-00835231, revealed a two-step binding mechanism, followed by covalent complex formation. The C145A M^pro^ mutant interacts with PF-00835231 approximately 100-fold less effectively. Nevertheless, the binding constant of PF-00835231 toward C145A M^pro^ is still good enough to inhibit the enzyme. Therefore, our results suggest that even noncovalent inhibitor binding due to a fine conformational fit into the active site is sufficient for efficient inhibition. A structure-based virtual screening and a subsequent detailed assessment of inhibition efficacy allowed us to select two compounds as promising noncovalent inhibitor leads of SARS-CoV-2 M^pro^.

## Introduction

Severe acute respiratory syndrome coronavirus 2 (SARS-CoV-2) was identified in 2020 as a novel member of the family *Coronaviridae* (genus *Betacoronavirus*) (Zhu et al., 2020a). This infectious agent causes coronavirus disease 2019 (COVID-19) and is a big threat to public health worldwide (Dong et al., 2020; Wu et al., 2020). The substantial achievements in the development of COVID-19 vaccines (Calina et al., 2020) as well as the design of neutralizing immunotherapeutics (Shang et al., 2020; Guo et al., 2021; Zhou et al., 2021) are promising milestones in the fight against the pandemic. On the other hand, attainment of stable protection against SARS-CoV-2-induced infection remains the most challenging problem of current life sciences. The development of effective drug discovery strategies and approaches, including the screening of small-molecule antivirals, necessitates in-depth knowledge about molecular and cellular mechanisms of coronavirus infection. This requires thorough research on specific targets of antiviral treatment at the molecular level (V’kovski et al., 2021). Directly acting and SARS-CoV-2–specific antiviral treatments are extremely limited today. A number of small-molecule compounds that may inhibit SARS-CoV-2 replication have been proposed (Zhu et al., 2020b; Khan et al., 2021; Wang et al., 2021). Nevertheless, only one directly acting antiviral nucleoside analog, namely remdesivir, which inhibits the SARS-CoV-2 RNA-dependent RNA polymerase, is currently approved (Pruijssers et al., 2020; Yin et al., 2020). The large 30 kb RNA genome of SARS-CoV-2 contains 13 open reading frames, two of which encode large polyproteins, processed by a 3C-like cysteine protease (main protease, M^pro^ or 3CL^pro^) at 11 sites and a papain-like cysteine protease (PL^pro^) at three sites, resulting in 16 nonstructural proteins, forming the replication complex. Both proteases are essential for the viral life cycle, making them alternate attractive targets for a therapeutic intervention (Cannalire et al., 2020; Rut et al., 2020; Ullrich and Nitsche, 2020; Qiao et al., 2021), and because of the more pronounced role of M^pro^ in the polyprotein processing, it is considered the primary SARS-CoV-2 enzyme target of directly acting antivirals.

**FIGURE 1:**
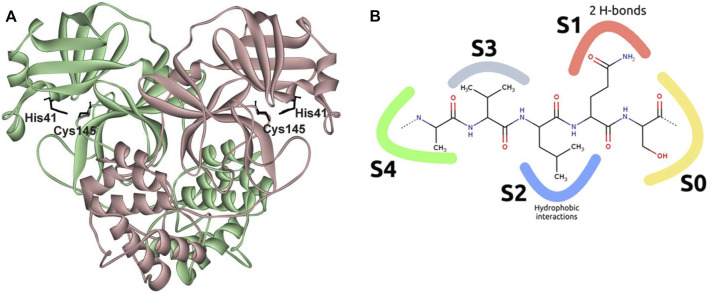
Structure of SARS-CoV-2 M^pro^. **(A)** Overview of the structure of an M^pro^ complex with PF-00835231 (Protein Data Bank [PDB] ID 6XHM (Hoffman et al., 2020); ligand not shown); **(B)** schematic representation of interactions of the active site with the substrate. The S0 subpocket is formed mostly by Asn142, Ser144, Cys145, and Leu27; S1 includes Phe140, His163, Glu166, and His172; S2 contains His41, Met49, Arg188, and Asp187; S4 includes Glu166, Leu167, Pro168, Gln189, and Ala191; and S3 is exposed on the outer surface of the active site.

The catalytically active form of M^pro^ is a homodimer with an extended substrate-binding site and a catalytic Cys145−His41 dyad (Ziebuhr and Siddell, 1999; Gadlage and Denison, 2010; Dai et al., 2020; Zhang et al., 2020; Noske et al., 2021) (Figure 1A). The active site consists of five subpockets: the S0 subpocket is formed mostly by Asn142, Ser144, Cys145, and Leu27; S1 includes Phe140, His163, Glu166, and His172; S2 contains His41, Met49, Arg188, and Asp187; S4 includes Glu166, Leu167, Pro168, Gln189, and Ala191; and S3 is exposed on the outer surface of the active site. M^pro^ selectively cleaves the −Y−Z−Leu−Gln↓−X sequence, where X is a small amino acid (Ser, Ala, or Gly), Y is a hydrophobic amino acid, and Z is a solvent-exposed amino acid residue (Figure 1B). Such substrate specificity is not shared by any known human protease, implying good potential for high specificity and a limited number of adverse effects of M^pro^-targeting antivirals (Zhang et al., 2020).

**FIGURE 2:**
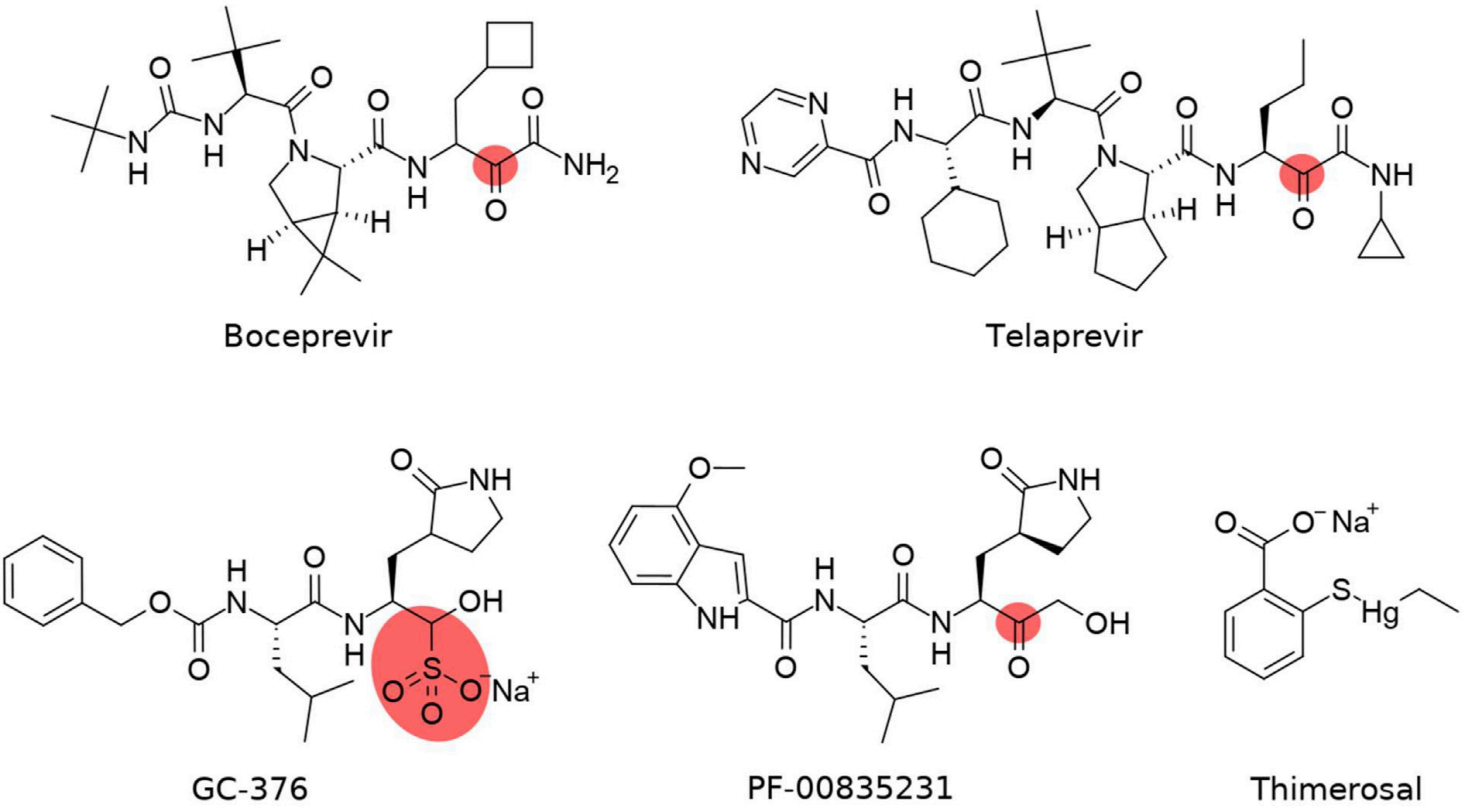
Structures of covalent inhibitors of SARS-CoV-2 M^pro^. The covalent-binding atom is highlighted. The sulphonate group in GC-376 is cleaved upon Cys145 binding.

Numerous small-molecule screening and drug repurposing programs involve M^pro^ as the target enzyme (Roe et al., 2021; Vandyck and Deval, 2021; Yang and Yang, 2021). The most prominent inhibitors appear to bear a reactive warhead that can form a covalent bond with M^pro^ residue Cys145 (several examples are given in Table 1 and Figure 2). Boceprevir and telaprevir, which are approved antiviral drugs targeting the hepatitis C NS3 protease, have emerged as SARS-CoV-2 M^pro^ inhibitors in numerous drug repurposing campaigns (Lang, 2007; Rotella, 2013). GC-376 has been specifically designed to target feline infectious peritonitis virus (FIPV) M^pro^ and has a potent antiviral activity against multiple coronaviruses, including MERS-CoV, FIPV, and subsequently SARS-CoV-2 (Kim et al., 2012; Kim et al., 2016; Pedersen et al., 2018; Vuong et al., 2021). One of the most promising compounds, PF-00835231, was initially designed in response to the previous coronavirus epidemic in 2003 as an inhibitor of SARS-CoV M^pro^ (Hoffman et al., 2020). Recent studies on this compound (Boras et al., 2021; de Vries et al., 2021) confirmed both the antiviral activity against SARS-CoV-2 and M^pro^ inhibition due to high conservation of the PF-00835231–binding site in M^pro^ between SARS-CoV and SARS-CoV-2. Thimerosal is an organometallic compound that possesses antibacterial properties due to its capacity to bind thiol groups in proteins, e.g., the catalytic cysteine of M^pro^, and came to the fore in an early drug repurposing screen (Geier et al., 2007; Coelho et al., 2020). Although there have been intensive efforts to develop M^pro^ inhibitors specific for SARS-CoV-2 (Jin et al., 2020a; Jin et al., 2020b; Hoffman et al., 2020; Ma et al., 2020; Yoshino et al., 2020), only PF-07304814 (a prodrug of PF-00835231) and its orally bioavailable analog PF-07321332 have reached clinical trials (Boras et al., 2021; Owen et al., 2021).

**TABLE 1:** M^pro^ 50% inhibitory concentration (IC_50_) for selected inhibitors.

Inhibitor	IC_50_, μM	References
Boceprevir	0.95	Baker et al. (2021)
	4.13	Ma et al. (2020)
	8.0	Fu et al. (2020)
	2.7	Anson et al. (2020)
	5.4	Ghahremanpour et al. (2020)
Telaprevir	15.2	Baker et al. (2021)
	10.7	Anson et al. (2020)
GC-376	0.026	Hung et al. (2020)
	0.030	Ma et al. (2020)
	0.15	Fu et al. (2020)
	0.17	Zhu et al. (2020c)
	0.62	Rathnayake et al. (2020)
	0.19	Vuong et al. (2020)
PF-00835231	0.007	Boras et al. (2021); de Vries et al. (2021)
	0.00027	Hoffman et al. (2020)
Thimerosal	0.6	Coelho et al. (2020)

According to reported data, the development of specific inhibitors with good binding parameters toward the SARS-CoV-2 main protease looks like a promising strategy against the COVID-19 pandemic. Thus, an effective screening and binding characterization pipeline for such compounds is in high demand (Zaidman et al., 2020). In the present work, we propose a screening platform for this purpose.

Here, we for the first time elucidated the key steps in the mechanism behind the enzyme–substrate and enzyme–inhibitor interactions that ensure specific binding and catalytic transformation. We employed a pre–steady-state kinetic approach. The stopped-flow kinetic analysis of sequential stages of model peptide binding and cleavage by M^pro^ allowed us to determine the rates of formation of the enzyme–substrate catalytic complex and peptide cleavage. It was found that the interaction of M^pro^ with PF-00835231 proceeds through two-step binding-complex formation with a subsequent chemical step of covalent bond formation. The strong reversible binding of PF-00835231 in the active site of C145A M^pro^ clearly indicates that potential inhibitors should have specific structural characteristics to finely fit into the pocket of the enzyme’s active site even without covalent bond formation. Next, structure-based virtual screening of small-molecule noncovalent inhibitors of M^pro^ was performed. Inhibition efficacy of these compounds was analyzed in the thermal shift assay and steady-state and pre–steady-state kinetic approaches, which enabled us to identify two new noncovalent inhibitors of SARS-CoV-2 M^pro^.

## Materials and Methods

### Protease Expression and Purification

A codon-optimized gene coding for full-length SARS-CoV-2 M^pro^ fused with the 6×His tag at the C terminus and with GST protein at the N terminus in plasmid vector pGEX6p (Zhang et al., 2020) was kindly provided by Prof. Rolf Hilgenfeld. The *M* ^*pro*^ gene was flanked by sequences of two protease sites for subsequent excision of native full-length M^pro^: a site recognized by M^pro^ for auto-excision (at the N terminus of the M^pro^ sequence) and a PreScission™ Pro site immediately before the 6×His tag for its removal, as described in (Zhang et al., 2020). The gene of the C145A M^pro^ mutant was generated by PCR-mediated site-directed mutagenesis using two overlapping primers and the M^pro^ pGEX6p vector as a template.

The full-length M^pro^ protein was overproduced in *E. coli* BL21 (DE3) and purified as described (Zhang et al., 2020) with minor modifications. Namely, the GST-M^pro^ fusion protein was subjected to self-processing during *E. coli* expression to prepare M^pro^ with the intact N terminus. M^pro^ fusion with the GST protein was employed to improve the M^pro^ yield and solubility. Next, M^pro^-His was purified by IMAC chromatography on TALON (Clontech) and treated with PreSсission™ Pro (M^pro^/PreSсission ratio 100:1) for 48 h at 4 °C for 6×His tag removal and obtaining M^pro^ with the intact C terminus. Then, a mixture of M^pro^ with PreSсission™ Pro (which contains a GST tag and His tag) was loaded on a GST-Sepharose (Amersham Biosciences) column and a TALON (Clontech) IMAC column, connected in tandem. Pure M^pro^ without tags was obtained in a flow-throw fraction. Western blot analysis with anti 6×His antibodies revealed that the 6×His tag was completely removed. M^pro^ was concentrated up to 10 mg/mL in 50 mM Tris (pH 7.5) and stored at −80°C. C145A M^pro^ mutant protein was obtained in the same way with an additional step of intact N-terminus generation, because the non-active C145A M^pro^ form cannot process itself during *E.coli* expression. For this purpose, IMAC-purified GST-C145A M^pro^His protein was treated by wild type M^pro^ (the GST-C145A M^pro^-His/M^pro^-ratio was 100:1), then the IMAC step was repeated to remove free M^pro^. Then C145A M^pro^His protein was subjected for PreSсission™ Pro cleavage and further procedures as described above.

### Peptide Substrate and Covalent Inhibitors

The kinetic assays were implemented using the FRET substrate (FRET-S), Dabcyl-KTSAVLQ↓SGFRKM-E(Edans)-NH_2_ (BPS Bioscience, United States), and standard covalent inhibitors GC-376, PF-00835231, boceprevir, telaprevir (Selleckchem, United States), and thimerosal (Serva). FRET-S contains a main-protease cleavage site (indicated by the arrow in the sequence above) and was utilized as the substrate in the FRET-based cleavage assay. Stock solutions of the inhibitors were prepared in DMSO (final concentration 5.0 mM).

### Virtual Screening of Noncovalent SARS-CoV-2 M^pro^ Inhibitors

Virtual screening was performed in July 2020 via the blind docking approach. The apo-structure of M^pro^ at room temperature [PDB ID 6WQF (Kneller et al., 2020)] was selected as the one representing the most physiologically relevant conditions. The protein structure was optimized in Chimera (Pettersen et al., 2004) using the *Dock Prep* tool: solvent molecules were deleted, while hydrogens (taking into account hydrogen bonds) and AMBER ff14SB charges (Maier et al., 2015) were added. Docking was performed in DOCK 6.9 (Allen et al., 2015). The grid box was generated to enclose the orthosteric binding site of chain A.

Docking-based virtual screening was performed on molecules previously selected from ZINC15 (Sterling and Irwin, 2015) as potential novel anticoronavirus compounds (hitlist *ZINCVS/novel.-smi_id_frq*) using the Generative Topographic Mapping approach (Horvath et al., 2020). Three-dimensional coordinates for the molecules were downloaded in MOL2 format from the ZINC15 website via the *“Search Many”* feature and used without further processing. Docked compounds were ranked by the values of *grid_score* for the best scored pose, and 22 of those with *grid_score* < −50 were designated as primary hits. They were grouped according to the molecule core and assessed for commercial availability, thereby leading to three cores available from a local supplier, Alinda (http://www.alinda.ru). The hitlist was then expanded to include compounds from the same classes with different substituents and *grid_score* < −45, resulting in an experimental assessment list of 10 compounds (Supplementary Table S1). Purchased compounds were used in experiments without further purification. Stock DMSO solutions were prepared with compound concentration of 5 mM.

### Thermal Shift Assay

Binding of inhibitors to SARS-CoV-2 M^pro^ was monitored by the TSA on a QIAGEN Rotor Gene Q Real Time PCR System. An M^pro^ solution (5 μM) was mixed with 50 μM inhibitor (or 1% DMSO), a dye (ProteOrange Protein Gel Stain; Lumiprobe, Russia; 5X in the final volume) in the buffer solution (20 mM HEPES, pH 6.5, 120 mM NaCl, 0.4 mM EDTA, 4 mM DTT, and 20% of glycerol) to attain the final volume of 25 μL. After 30 min incubation at 30 °C, each sample was heated to 95 °C with 0.05 °C/s increment, and fluorescence was monitored in the green channel (λ_ex_/λ_em_ = 470/510 nm). T_m_ was calculated as the maximum of the first derivative of the fluorescence signal in Origin 2017 software (OriginLab).

### Steady-State Kinetic Assay

For these assays, 160 nM M^pro^ in reaction buffer (20 mM Tris-HCl pH 7.3, 100 mM NaCl, 1.0 mM EDTA, and 1.0 mM DTT) was incubated with or without a tested compound at various concentrations for 30 min at 30 °C. The reaction was initiated by the addition of FRET-S (0–80 μM) in reaction buffer. Substrate is cleaved by M^pro^ generating a product containing a free Edans group. The dequenching of fluorescence by the cleavage of the substrate catalyzed by M^pro^ was monitored at 460 nm with excitation at 360 nm on a Thermo Scientific Varioscan plate fluorimeter. The concentration of a fluorescent product was determined according to the calibration curve of free Edans fluorescence covering a concentration range of 0.1–20.0 μM. Initial rates of FRET-S cleavage by M^pro^ at each inhibitor concentration were computed from reaction kinetic curves of M^pro^ activity at several substrate concentrations. Kinetic constants (V_max_ and *K*_M_) were derived by fitting the data to the Michaelis–Menten equation, V = V_max_ × [S]/(*K*_M_ + [S]). After that, *k*_cat_ was calculated according to the equation *k*_cat_ = V_max_/[E].

For the preliminary screening of noncovalent M^pro^ inhibitors, 160 nM M^pro^ was incubated with 80 μM inhibitor (or 1.6% DMSO) for 30 min at 30 °C in reaction buffer, and then 16 μM FRET-S was added to initiate the reaction (final volume 20 μL). The fluorescence signal of the reaction was monitored for 2 h at λ_ex_/λ_em_ = 355/460 nm (Thermo Scientific Fluoroskan FLash fluorimeter). The initial rate was calculated by linear regression for the first 10 min of the kinetic progress curves. Residual activities were computed by dividing the initial velocity in the presence of an inhibitor by the initial rate in its absence (DMSO control).

### Stopped-Flow Measurements

Stopped-flow measurements with fluorescence detection were carried out using a SX.20 stopped-flow spectrometer (Applied Photophysics Ltd., United Kingdom) equipped with a 150-W Xe arc lamp and an optical cell with 2 mm path length. The dead time of the instrument is 1.0 ms. For the analysis of enzyme–substrate interactions, the FRET-S substrate modified with the dye–quencher pair Edans/Dabcyl was utilized. The fluorescence of Edans was excited at λ_ex_ = 340 nm and monitored at λ_em_ > 435 nm as transmitted by filter GG-435 (Schott, Mainz, Germany). The binding of M^pro^ to PF-00835231 was monitored by means of changes in intrinsic fluorescence intensity of the inhibitor. The excitation wavelength was 300 nm, and the emission was monitored using long-pass wavelength filters at λ_em_ > 370 nm (Corion filter LG-370).

The enzyme was placed in one of the instrument’s syringes and rapidly mixed in the reaction chamber with the substrate, inhibitor, or a substrate/inhibitor mixture from another syringe. The concentration of FRET-S in all the experiments was 2.5 μM, while concentrations of M^pro^ or its C145A mutant were varied from 0.1 to 3.0 μM. The reported concentrations of reactants are those in the reaction chamber after the mixing. All experiments were conducted at 25 °C in the reaction buffer.

### Global Fitting of the Stopped-Flow Data

Kinetic simulation of the time course of appearance and disappearance of various reaction intermediates was done by solving a system of differential equations in the DynaFit software (BioKin, Pullman, WA) (Kuzmic, 1996) as described before (Zakharova et al., 2009; Zakharova et al., 2017). The fast kinetic analysis combined with fluorimetry detection of conformational changes is a powerful method that may provide detailed information about mechanisms of enzyme–substrate interaction (Kuznetsov and Fedorova, 2016; Kuznetsov et al., 2017; Kladova et al., 2019; Kuznetsova et al., 2020; Kuznetsov and Fedorova, 2020). This approach is based on fluorescence intensity variation in the course of the reaction owing to sequential formation and subsequent transformation of the enzyme–substrate complex. The stopped-flow fluorescence traces were directly fitted to the fluorescence intensity at any reaction time point as the sum of background fluorescence and fluorescence intensity values of each intermediate complex that contribute to the signal.

The software performs numerical integration of a system of ordinary differential equations with subsequent nonlinear least-squares regression analysis. In the evaluated mechanisms, except for the first bimolecular step, all other reactions are first-order. In the fits, we optimized all relevant rate constants for the forward and reverse reactions as well as specific molar response factors for all intermediate complexes.

During the data processing, the kinetic information was obtained from the temporal behavior of the fluorescence intensity, not from the amplitudes of the specific signal contributions. The response factors for different states resulting from the fits were not used for determining equilibrium constants but rather provided additional information on fluorescence intensity variations in different states of the complex.

## Results and Discussion

### Interaction of Wild-Type M^pro^ and C145A M^pro^ with the Substrate

To thoroughly characterize the kinetic mechanism underlying catalytic cleavage of a peptide, pre–steady-state kinetic assays based on the Förster resonance energy transfer (FRET) effect were performed. The Dabcyl-KTSAVLQSGFRKM-E(Edans)-NH_2_ peptide armed with a dye–quencher pair was used for FRET measurements (Zhu et al., 2020c; Hoffman et al., 2020). FRET analysis could reveal changes in the distance between the dye and quencher in the processes of peptide penetration into the active site and formation of specific contacts between the side chains of the substrate and the respective binding cavities—that subsequently result in the catalytic state, hydrolysis of the peptide bond, and the release the products.

**FIGURE 3:**
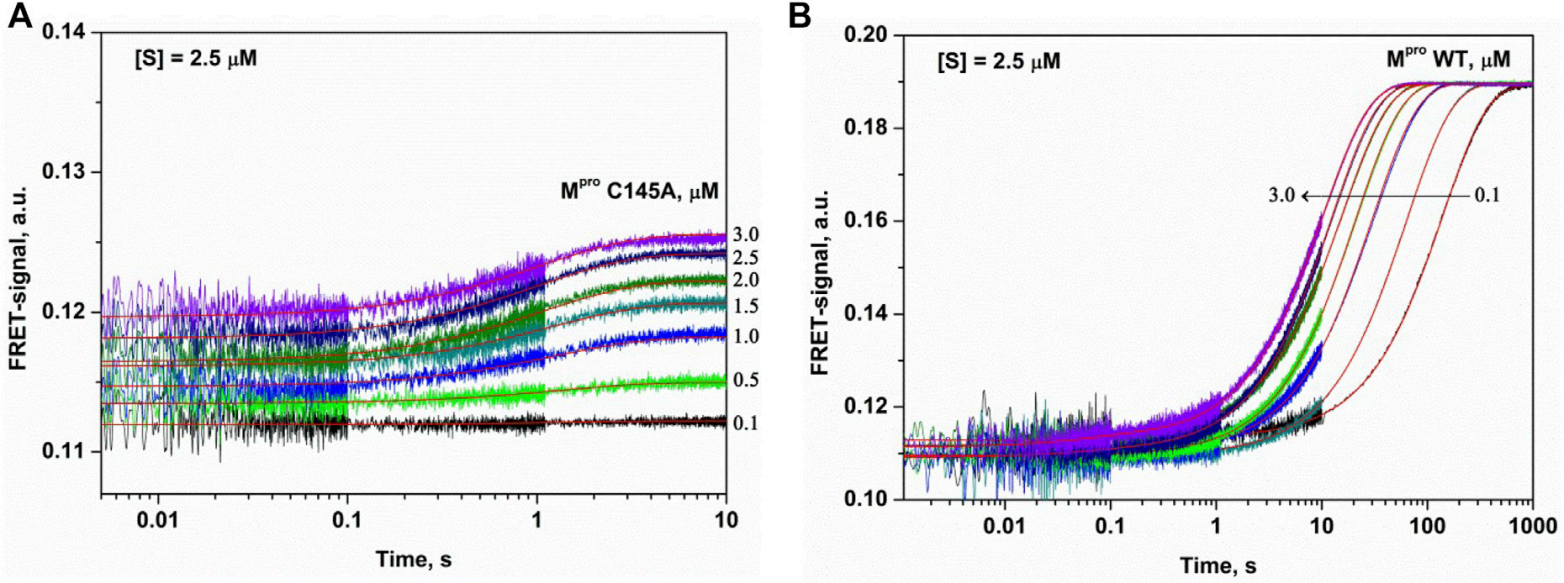
Experimental and theoretical (smooth curves) kinetic curves for the FRET signal changes during the interaction of C145A M^pro^ **(A)** or WT M^pro^ **(B)** with FRET-S. The FRET-S concentration was 2.5 µM, and the enzyme concentration is indicated in the panel.

**SCHEME 1:**
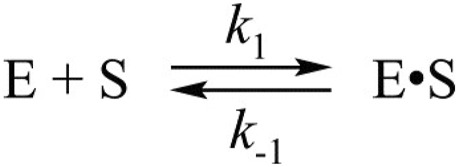
The kinetic mechanism of the interaction between C145A M^pro^ and FRET-S. E: C145A M^pro^, S: substrate, E•S: enzyme–substrate complex.

The C145A substitution led to complete elimination of the catalytic activity of the protease owing to a loss of the catalytic thiol group. The interaction of C145A M^pro^ with the substrate can lead only to its binding and formation of a preincision complex. Indeed, as shown in Figure 3A, the FRET signal during the interaction of C145A M^pro^ with the substrate increased up to time point of 5 s. It is possible that the increase in the FRET signal reflects increased distance between the fluorogenic Edans residue and quenching Dabcyl residue owing to peptide stretching in the active site of the protease. An analysis of the kinetic curves suggested that the minimal kinetic mechanism of the interaction between the catalytically inactive mutant and FRET substrate involved one-step equilibrium binding (Scheme 1). The rate constants for the forward and reverse reactions are given in Table 2.

**TABLE 2:** Rate constants of the interaction of WT M^pro^ and C145A M^pro^ with the substrate.

	WT	C145A
*k*_1_, M^−1^×s^−1^	(0.26 ± 0.05) × 10^6^	(0.28 ± 0.02) × 10^6^
*k*_-1_, s^−1^	0.9 ± 0.1	0.18 ± 0.01
*K*_1_, M^−1^	(0.3 ± 0.1) × 10^6^	(1.6 ± 0.1) × 10^6^
*k*_cat_, s^−1^	0.29 ± 0.03	–
*K*_D_, M	3.3 × 10^–6^	0.6 × 10^–6^
*K*_M_, M	4.6 × 10^–6^	–

*K*_1_ = *k*_1_ */k*_−1_, *K*_D_, 1*/K*_1_, *K*_M_ = (*k*_−1_ + *k*_cat_)*/k*_1_.

**SCHEME 2:**
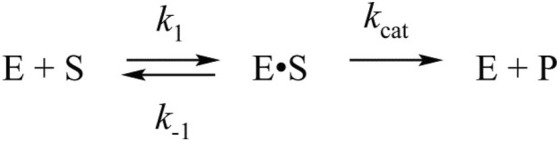
The kinetic mechanism of the interaction between WT M^pro^ and FRET-S. E: WT M^pro^, S: substrate, E•S: enzyme-substrate complex, P: product.

The interaction of WT M^pro^ with the substrate was slower and proceeded up to 100 s but caused a high amplitude increase in the FRET signal (Figure 3B). Such a growth of the FRET signal most likely reflects a release of the incised peptide products from the complex with the enzyme. Taking into account that the catalytically inactive mutant form revealed one-step binding mechanism, we assumed two-step mechanism of product formation when WT protease interacts with the FRET substrate. Indeed, the kinetic curves were satisfactorily described by Scheme 2, containing one equilibrium stage of substrate binding and one irreversible step of hydrolysis and release of the reaction products (Table 2).

It should be noted that the rate constants of the substrate binding were not affected by the C145A substitution. Nevertheless, the total binding constant was approximately fivefold higher in the case of the C145A variant owing to a decrease in *k*_−1_. These findings suggested that the Cys145 residue influenced the stability of the enzyme–substrate complex but did not affect the rate of peptide binding.

**FIGURE 4:**
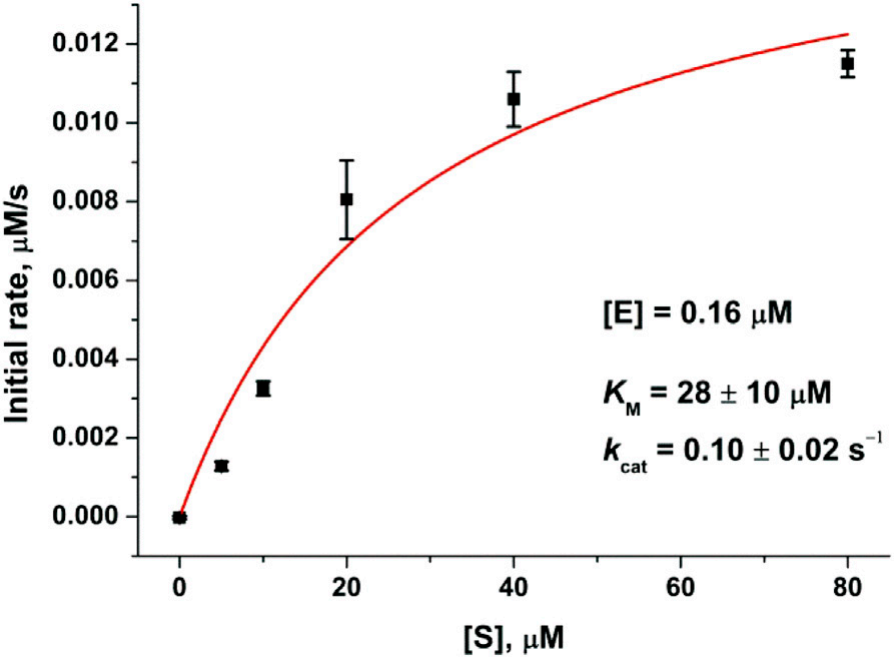
Dependence of the initial rate of substrate cleavage by SARS-CoV-2 M^pro^. Each data point is an average from at least three independent experiments, the values are presented as the mean ± SE.

To verify the kinetic scheme and the rate constants calculated by the global fitting procedure, we determined steady-state reaction parameters: Michaelis constant *K*_M_ and catalytic reaction rate constant *k*_cat_ (Figure 4). The Michaelis constant *K*_M_ calculated from the elementary kinetic constants via the formula *K*_M_ = (*k*_cat_ + *k*_*–1*_)/*k*_1_ (4.6 μM, Table 2) was three- to sixfold lower than the value obtained by the steady-state analysis (28 μM, Figure 4) or reported earlier [14 μM (Hoffman et al., 2020)]. Catalytic constants *k*_cat_ were similar between the stopped-flow and steady-state analyses, suggesting that the fast kinetic pre–steady-state approach allowed us to determine relevant characteristics of the enzymatic reaction. Fitting of the steady-state data by the Hill equation (*n* = 2, Supplementary Figure S1) also provides similar constant K_50_ of 50% enzyme saturation (15.7 μM). The determined Hill coefficient indicates positive cooperativity and is in good agreement with the reported data (Lee et al., 2020; Vuong et al., 2020). However, complication of the pre–steady-state kinetic mechanism (Scheme 1) up to the two-substrate binding model does not provide a better fit of the experimental data, supporting independent action of each active site of the enzyme in the pre–steady-state conditions.

### Interaction of WT M^pro^ and C145A M^pro^ With PF-00835231

**FIGURE 5:**
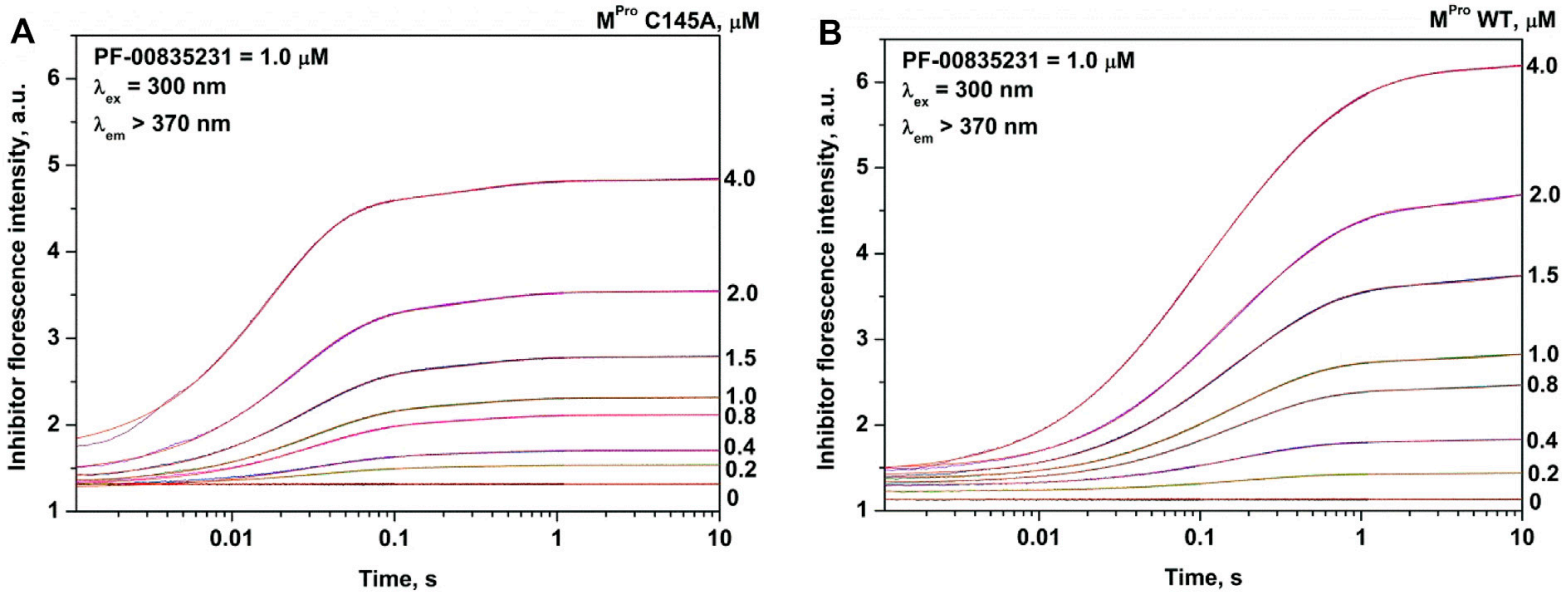
Experimental and theoretical (red) kinetic curves of changes in PF-00835231 fluorescence intensity during the interaction with C145A M^pro^ **(A)** or WT M^pro^ **(B)**. The concentration of PF-00835231 was 1.0 µM, and the enzyme concentration is shown in the panel. Substantial overlap of experimental and theoretical kinetic curves indicates good fitting quality and masks visual differences between experimental and theoretical traces.

**SCHEME 3:**
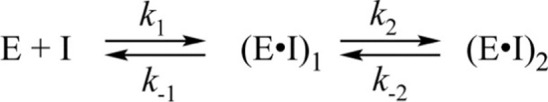
The kinetic mechanism of the interaction between C145A M^pro^ and PF-00835231. E: C145A M^pro^, I: PF-00835231, (E•I)_i_: enzyme–inhibitor noncovalent complexes.

**FIGURE 6:**
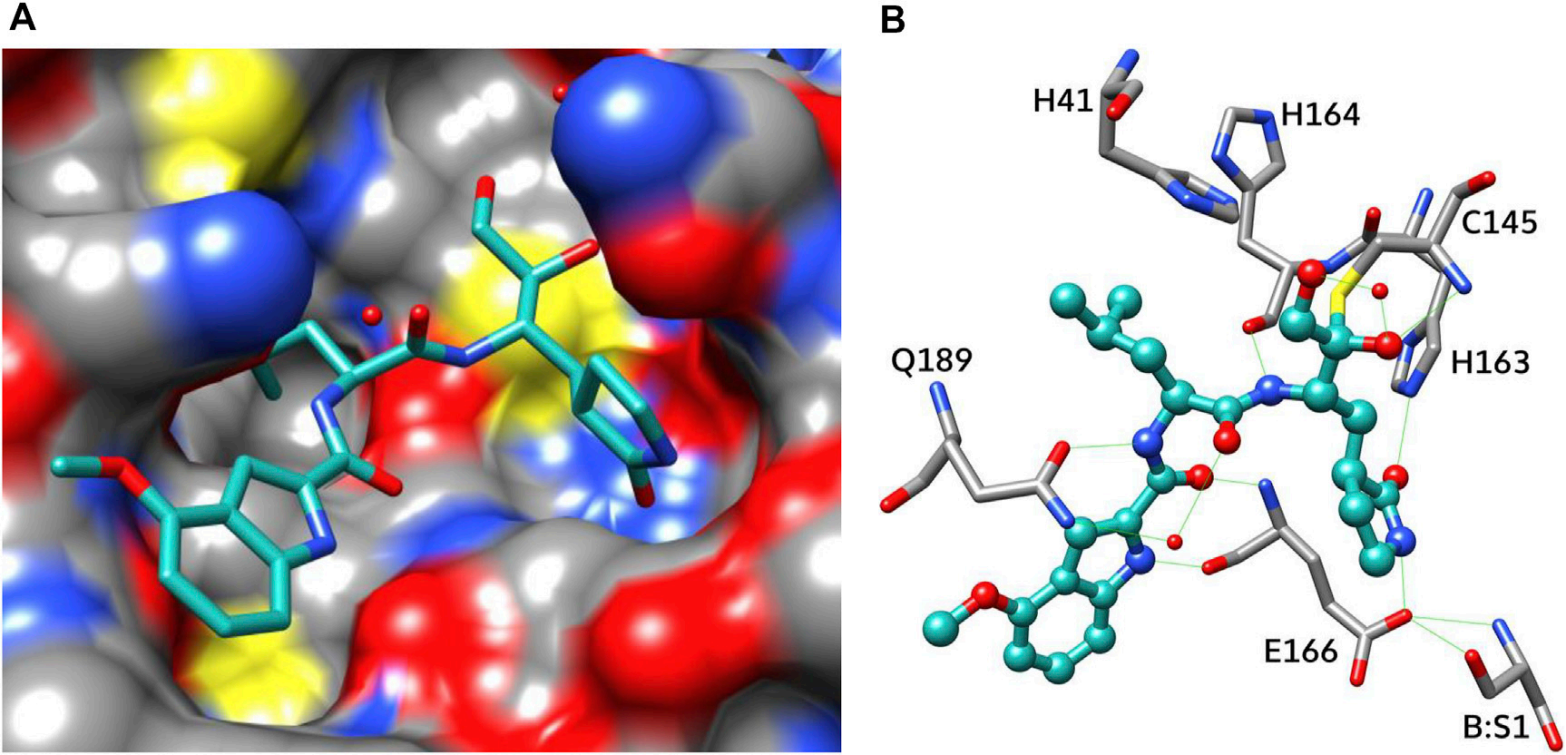
Binding mode of PF-00835231 (PDB ID 6XHM): surface representation **(A)** and an interaction scheme **(B)**. The inhibitor molecule is shown as a ball-and-stick model, water molecules as red balls, and hydrogen bonds as green lines.

The analysis of the PF-00835231 binding kinetics in the course of its interaction with WT or C145A M^pro^ was performed by the stopped-flow technique with detection of intrinsic florescence intensity of PF-00835231. The association of C145A M^pro^ with PF-00835231 led to a two-phase increase in the fluorescence intensity up to time point 2 s (Figure 5A). The kinetic curves were satisfactorily described by Scheme 3, which contains two equilibrium stages (Table 3). It is likely that after the formation of the initial complex, there is an additional step of formation of specific interactions between inhibitor moieties and enzyme cavities resulting in full insertion of the inhibitor molecule into the active site of the protease (Figure 6). Of note, the affinity of C145A M^pro^ for PF-00835231 even without the covalent binding was 2.5 μM, strongly supporting perfect complementarity between the inhibitor and the active site, as first revealed by X-ray crystallography (Hoffman et al., 2020).

**TABLE 3:** Rate constants for the interaction of WT M^pro^ or C145A M^pro^ with PF-00835231.

	WT	C145A
*k*_1_, M^−1^×s^−1^	(0.35 ± 0.09)×10^6^	(6.6 ± 0.2)×10^6^
*k*_-1_, s^−1^	12.4 ± 0.6	22 ± 0.2
*K*_1_, M^−1^	0.028 ± 0.008	0.30 ± 0.02
*k*_2_, s^−1^	0.90 ± 0.18	1.0 ± 0.2
*k*_-2_, s^−1^	2.4 ± 0.1	2.8 ± 0.2
*K*_2_	0.37 ± 0.07	0.35 ± 0.09
*k*_chem_, s^−1^	0.17 ± 0.07	–
*K*_D_, M	(29 ± 11)×10^–6^	(2.5 ± 0.3)×10^–6^

*K*_1_ = *k*_1_ */k*_−1_, *K*_2_ = *k*_2_ */k*_−2_, *K*_D_, 1*/K*_ass_, *K*_ass_ = *K*_1_ + *K*_1_ × *K*_2_.

**SCHEME 4:**
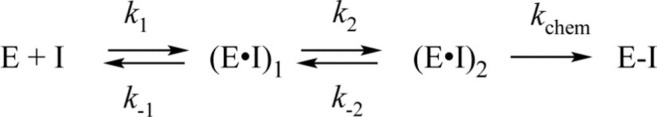
The kinetic mechanism of the interaction between WT M^pro^ and PF-00835231. E: WT M^pro^, I: PF-00835231, (E•I)_i_: enzyme–inhibitor noncovalent complexes, E–I: enzyme–inhibitor covalent complex.

The process of interaction of WT M^pro^ with PF-00835231 is slower (up to time point 10 s) and induces a greater increase in the fluorescence intensity (Figure 5B). It should be noted that in the case of the WT enzyme, the initial phase of the fluorescence intensity increase was significantly slower as compared with C145A M^pro^, indicating that the Cys145 residue must play an important role in the formation of the initial complex. As soon as the binding of the inhibitor to C145A M^pro^ went through two steps, the kinetic curves for the WT enzyme could be described by Scheme 4, which contains two equilibrium steps and one irreversible step (Table 3).

The value of rate constant *k*_1_ revealed that the formation of the initial complex is approximately 20-fold faster for C145A M^pro^, eventually yielding a 10-fold difference in the equilibrium binding constant between the WT and C145A enzymes. On the other hand, rate constants of the second binding step were very similar between the two enzymes, indicating that the specific interaction of the inhibitor and enzyme is independent of the Cys145 residue. The rate constant for the formation of the covalent bond with the inhibitor was similar to the catalytic constant of peptide cleavage (Tables 2 and 3).

Overall, it can be concluded that noncovalent interaction of WT M^pro^ and the PF-00835231 inhibitor is not as strong as expected from the nanomolar range of the inhibition constants reported in several studies (Hoffman et al., 2020; Boras et al., 2021; de Vries et al., 2021). Nevertheless, the high efficiency of PF-00835231 is explained by the subsequent covalent modification of the enzyme; this modification significantly stabilizes the enzyme–inhibitor complex.

### Virtual Screening and characterization of Noncovalent M^pro^ Inhibitors

**FIGURE 7:**
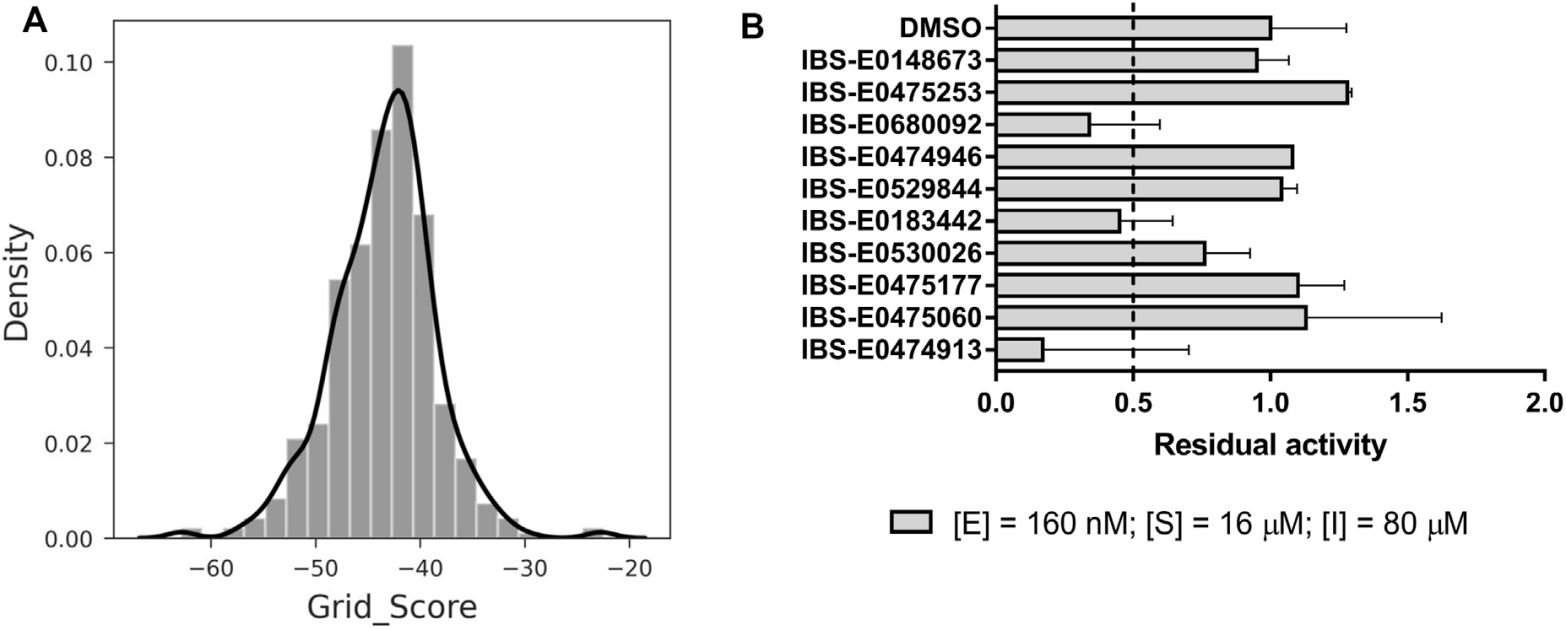
Structure-based virtual screening and an assessment of inhibition efficacy. **(A)** Docking score distribution for Generative Topographic Mapping hits. More negative values correspond to better scores. **(B)** Preliminary screening of the compounds for M^pro^ inhibition.

A two-step virtual screening procedure was carried out to prioritize commercially available small-molecule compounds as potential M^pro^ inhibitors. At the first step, an antiviral chemical space (Nikitina et al., 2019) analysis was performed using the Generative Topographic Mapping approach (Horvath et al., 2020). Of 800 million ZINC compounds, 574 were predicted as hits potentially possessing an anticoronaviral activity. Items of this hitlist were then docked into the active-site cavity of room temperature M^pro^ crystal structure 6WQF (Kneller et al., 2020), and the best compounds were selected according to the scoring function (Figure 7A, Supplementary Table S1). Ten compounds with grid scores less than −50 were designated as primary hits and subjected to the initial screening of M^pro^ inhibition (Figure 7B). Three compounds—IBS-E0680092, IBS-E0183442, and IBS-E0474913—showed more than 50% enzyme inhibition, whereas IBS-E0530026 manifested a less prominent inhibitory activity.

**FIGURE 8:**
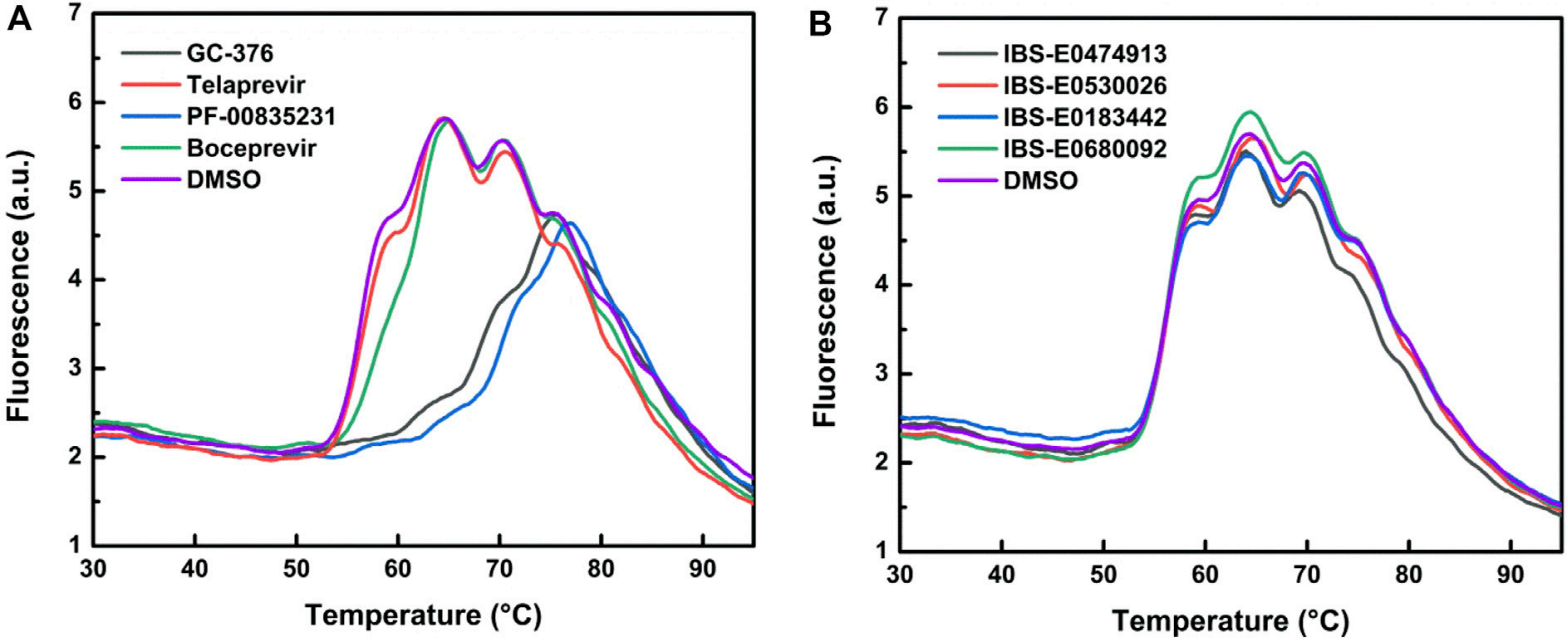
Melting curves of WT M^pro^ in the absence and presence of covalent **(A)** or noncovalent **(B)** inhibitors.

These four compounds were chosen for a more detailed comparison with covalent inhibitors of M^pro^. First of all, the protein stabilization by inhibitor binding was compared using thermal shift assay (TSA). TSA allows a direct comparison of protein binding efficiency between small-molecule compounds. A substantial change of protein melting temperature (T_m_) may be an indicator of tight binding (Figure 8; Table 4). The binding of covalent inhibitors GC-376, PF-00835231, and boceprevir caused changes of protein T_m_, pointing to strong stabilization of the enzyme molecule in the covalent complex with these compounds. On the other hand, the interaction of telaprevir and all noncovalent inhibitors did not result in T_m_ changes, suggesting that the binding of these compounds with the enzyme is less efficient.

**TABLE 4:** Changes in the M^pro^ melting temperature upon the inhibitor binding, and inhibition constants of the tested compounds.

Compound	T_m_ (°C)	ΔT_m_ (°C)	*K*_i_, μM
PF-00835231	70.25	14.00	0.004
GC-376	68.25	12.00	0.03
Boceprevir	62.00	5.75	3.3
Telaprevir	56.25	0.0	15.6
IBS-E0680092	56.25	0.0	20.7
IBS-E0183442	56.25	0.0	26.3
IBS-E0474913	56.25	0.0	28.6
IBS-E0530026	56.25	0.0	—
DMSO (control)	56.25	0.0	—

Steady-state analysis of the inhibition efficacy of all the tested compounds revealed (Table 4) that the thermal shift is directly related to inhibition constant *K*_i_. Indeed, the decrease of ΔT_m_ correlated with an increase of the inhibition constant. Moreover, the thermal shift was negligible when *K*_i_ approached ∼10–15 μM. Furthermore, the elimination of the M^pro^ covalent binding with PF-00835231 by means of the C145A mutation also strongly decreased the change in T_m_ (to ∼2.2°C) as compared to the covalently bound WT adduct (∼14.0°C), consistently with the pre-steady-state data, which yielded an inhibition constant of 2.5 μM (Table 3).

### Interaction of WT M^pro^ with a Substrate in the Presence of Inhibitors

**FIGURE 9:**
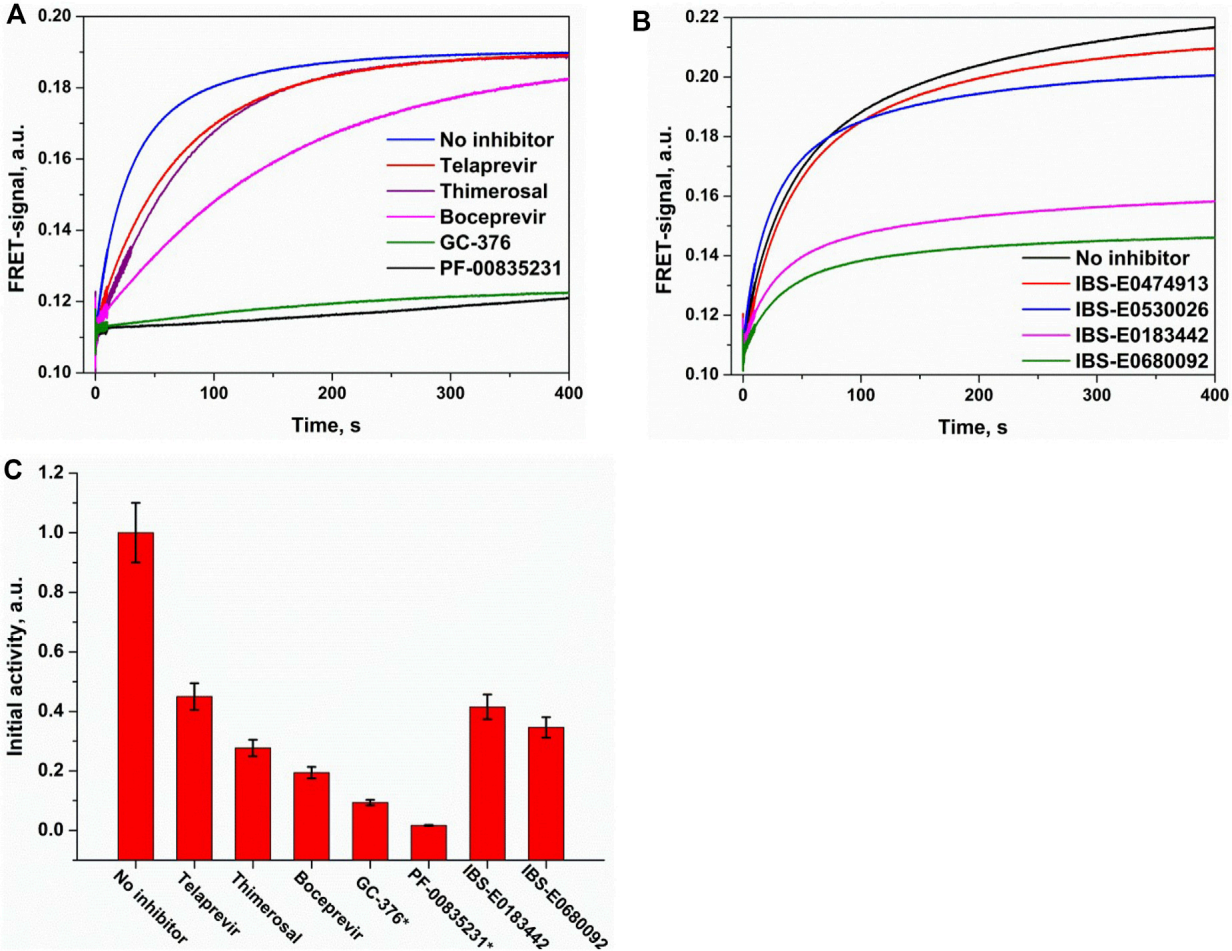
Comparative analysis of M^pro^ inhibition by covalent **(A)** and noncovalent **(B)** inhibitors. **(C)** Residual enzymatic activity calculated from the initial slope of kinetic curves. The activity of free enzyme was normalized to 1.0. FRET-S and enzyme concentrations were 2.5 µM, and the inhibitor concentration was 25 µM except for the compounds marked with an asterisk (2.5 µM).

The efficiency of enzymatic hydrolysis of the substrate by M^pro^ in the presence of a standard inhibitor (boceprevir, telaprevir, GC-376, PF-00835231, or thimerosal; Figure 9A) or a new inhibitor from the virtual screening (Figure 9B) was determined by comparison of the kinetics of the substrate cleavage. The protease was mixed with an inhibitor and kept on ice for 5 min to obtain an enzyme–inhibitor complex. After that, the substrate hydrolysis was initiated by stopped-flow fast mixing of this complex with FRET-S, and the signal was monitored. To estimate the remaining enzymatic activity, the initial slope of the FRET signal increase was calculated (Figure 9C). A comparison of the obtained data allowed us to conclude that both GC-376 and PF-00835231 were the most potent covalent inhibitors of M^pro^ among the tested ketone-based and organometallic compounds. Boceprevir showed an intermediate level of activity, whereas telaprevir and thimerosal were on the lowest activity tier, in line with the specificity of the design approach and binding-site complementarity for each of these compounds. Indeed, GC-376 and PF-00835231 were specifically designed as coronavirus M^pro^ inhibitors; boceprevir is less complementary to the binding site but still has a similar molecular size. On the other hand, telaprevir is much larger and engages in fewer specific interactions although it is still able to form a covalent bond with Cys145. Thimerosal, on the contrary, is substantially smaller and does not form specific interactions in the binding site; these features reduce the inhibitory mechanism of thimerosal to pure Cys145 blockage.

An assessment of relative efficacy of M^pro^ inhibition by the noncovalent binders revealed that two of them, IBS-E0183442 and IBS-E0680092, significantly inhibit the enzymatic activity, albeit less potently than the covalent inhibitors. This observation supports possible further inhibitor optimization via introduction of additional chemical groups to improve specific interactions as well as indicates that such a pre–steady-state analysis may be a good platform for rapid low-cost screening of small molecule compounds to reveal their inhibitory potential.

## Conclusion

Even though the SARS-CoV-2 main protease is an attractive target for a therapeutic intervention into COVID-19, only an extremely small number of compounds are currently known with inhibitory properties toward this enzyme. Therefore, the design of specific inhibitors as well as the development of effective screening systems for such compounds are urgently needed. In the present work, for the first time, we report a pre–steady-state kinetic analysis of sequential stages of a model peptide’s binding and cleavage by WT M^pro^. The fast-kinetics approach enables determining the rates of formation of an enzyme–substrate catalytic complex and rates of peptide cleavage by M^pro^ on the basis of the FRET effect. An interaction of the catalytically inactive mutant enzyme (C145A M^pro^) with the same substrate revealed that the enzyme normally induces conformational changes in the peptide during the complex formation. Our findings suggest that the binding of a peptide substrate in the active site of this protease proceeds through a single reversible stage in the kinetic scheme. A collision of the enzyme and peptide substrate rapidly gives rise to the catalytic complex in which site-specific cleavage of the peptide takes place.

The efficiency of enzymatic hydrolysis of the peptide substrate by M^pro^ in the presence of one suitable inhibitor was determined by a pre–steady-state kinetic analysis. In this study, the most promising covalent inhibitors of SARS-CoV-2 M^pro^ such as PF-00835231, GC-376, boceprevir, and telaprevir were tested. Thimerosal was also used because it is an organometallic binder of the catalytic cysteine of M^pro^. As expected, among all the tested covalent inhibitors, PF-00835231 was the most effective. It turned out that the interaction of M^pro^ with PF-00835231 involves two steps of reversible binding-complex formation with subsequent covalent binding step (Scheme 4). In this scheme it is likely that after the formation of the initial complex, there is an additional step of formation of specific interactions between the inhibitor and the enzyme pocket, resulting in the proper placement of the inhibitor in the active site of the protease. The irreversible step of Scheme 4 corresponds to the covalent bond formation between Cys145 of the enzyme and the inhibitor molecule. Indeed, the interaction of PF-00835231 in the active site of C145A M^pro^, which is incapable of a covalent binding with this compound, revealed that there are only two reversible steps of binding-complex formation. This result meant that potential inhibitory compounds should have certain pharmacophoric features for a fine conformational fit and must engage in specific interactions in the active-site pocket of the enzyme rather than contain specific warheads forming a covalent bond with the protein. Then, we performed a structure–guided selection, which yielded four small molecule-weight promising noncovalent inhibitors of M^pro^. The inhibition efficacy of these compounds was analyzed by a TSA and steady-state and pre–steady-state kinetic approaches, which helped us to identify two new noncovalent inhibitors of SARS-CoV-2 M^pro^.

Overall, we developed a platform for low-cost rapid quantitative estimation of the type and magnitude of inhibition for prospective inhibitors of the SARS-CoV-2 main protease. The analysis of kinetics using the WT enzyme together with its catalytically inactive mutant, C145A, enabled us to identify the mechanism of action of the inhibitors and gave an opportunity to hypothesize the therapeutic potential of a model drug. This approach allows us to work with various mutants of the enzyme as well as different types of inhibitors (specific to allosteric or active sites) and thus may be regarded as a technique supported by proof of concept in a target-based drug assessment prior to preclinical studies. The high sensitivity of the method and its ability to provide precise quantitative data will help us to discriminate relevant compounds by their kinetic properties. If the dynamics of the interaction of an enzyme with an inhibitor are crucial for the therapeutic potential of the drug, then the proposed technique will give a unique opportunity to accept/reject the compounds selected by means of molecular docking simulations.

## Data Availability

The original contributions presented in the study are included in the article/Supplementary Material. Further inquiries can be directed to the corresponding authors.
