# Supplementary material for: Pre-Steady-State Kinetics of the SARS-CoV-2 Main Protease as a Powerful Tool for Antiviral Drug Discovery
Source: Front Pharmacol. 2021 Dec 6;12:773198. doi: 10.3389/fphar.2021.773198 (PMC8686763; doi:10.3389/fphar.2021.773198)
Supplement: Supplementary file 1 [file DataSheet1.docx]

**
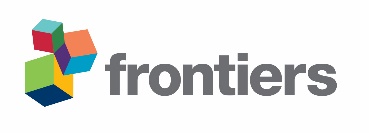
**

Supplementary Material

Pre-steady state kinetics of SARS-CoV-2 main protease as a powerful tool for antiviral drug discovery

Maria Yu. Zakharova,^1,2,†^ Alexandra A. Kuznetsova,^3,†^ Viktoria I. Uvarova,^4^ Anastasiia D. Fomina,^4,5^ Liubov I. Kozlovskaya,^4,5^ Elena N. Kaliberda,^1^ Inna N. Kurbatskaia,^1^ Ivan V. Smirnov,^1,5^ Anatoly A. Bulygin,^3^ Vera D. Knorre,^1^ Olga S. Fedorova,^3^ Alexandre Varnek,^7^ Dmitry I. Osolodkin,^4,5,6^ Aydar A. Ishmukhametov,^4,6^ Alexey M. Egorov,^4,5,*^ Alexander G. Gabibov,^1,5,8,^* and Nikita A. Kuznetsov^3,9,^*

^1^Institute of Bioorganic Chemistry Russian Academy of Sciences, Moscow 117997, Russian Federation;

^2^Pirogov Russian National Research Medical University, Moscow 117997, Russian Federation

^3^SB RAS Institute of Chemical Biology and Fundamental Medicine, Novosibirsk 630090, Russian Federation;

^4^FSASI “Chumakov R&D IBP RAS” (Institute of Poliomyelitis), Moscow 108819, Russian Federation

^5^Lomonosov Moscow State University, Moscow 119991, Russian Federation.

^6^Sechenov First Moscow State Medical University, Moscow 119991, Russian Federation

^7^Laboratoire de Chémoinformatique, UMR 7140 CNRS, Université de Strasbourg, Strasbourg 67000, France

^8^Department of Biology and Biotechnology, Higher School of Economics, Moscow, Russian Federation.

^9^Department of Natural Sciences, Novosibirsk State University, Novosibirsk 630090, Russian Federation.

^†^These authors contributed equally to this work.

^*^Correspondence may be addressed to A.M.E. (alex.m.egorov@gmail.com, tel. +7 (903) 969-0236), A.G.G. (gabibov@mx.ibch.ru, tel. +7 (495) 727-3860) or N.A.K. (nikita.kuznetsov@niboch.nsc.ru, tel. +7-383-3635174).

Table S1. Virtual screening hits purchased from Alinda.

| Compound | Structure | Grid score | Residual activity @ 80 uM | Best scored pose |
| --- | --- | --- | --- | --- |
| IBS-E0474913 | 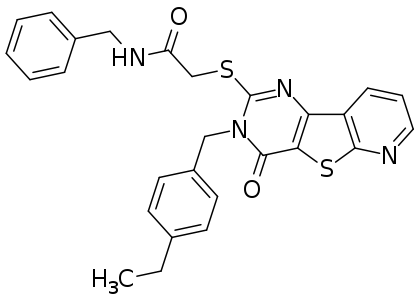 | -57.06 | 0.17 | 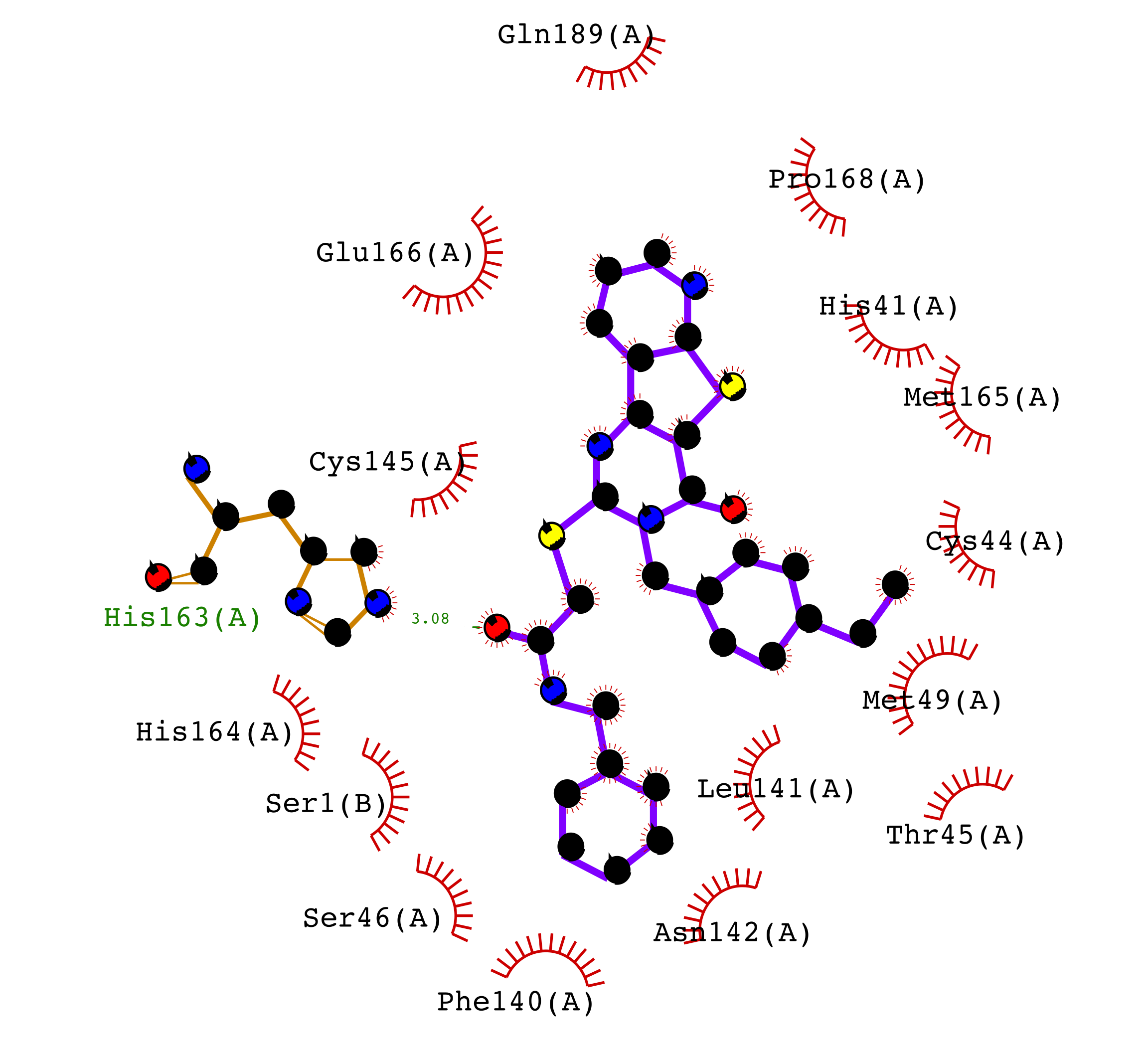 |
| IBS-E0475060 | 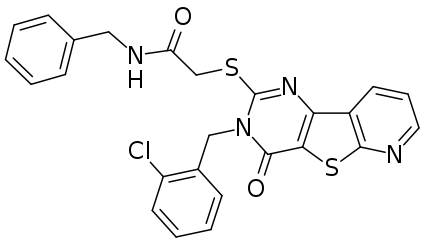 | -55.46 | 1.13 | 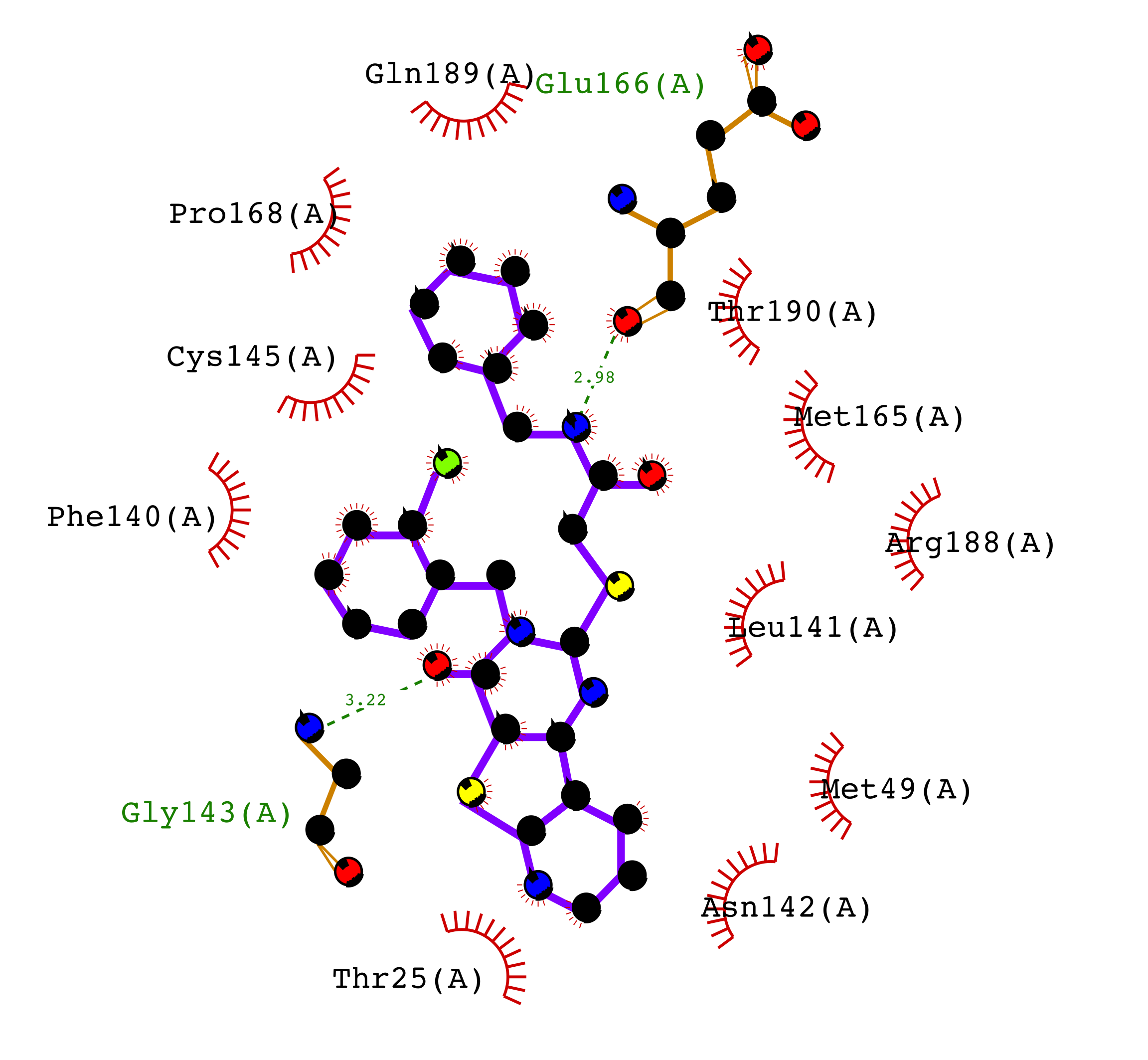 |
| IBS-E0475177 | 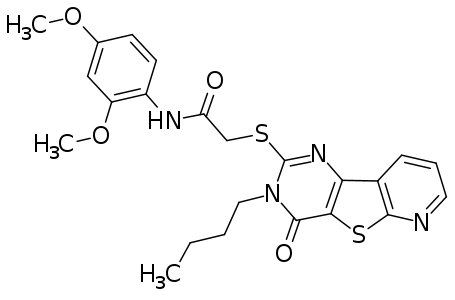 | -53.22 | 1.10 | 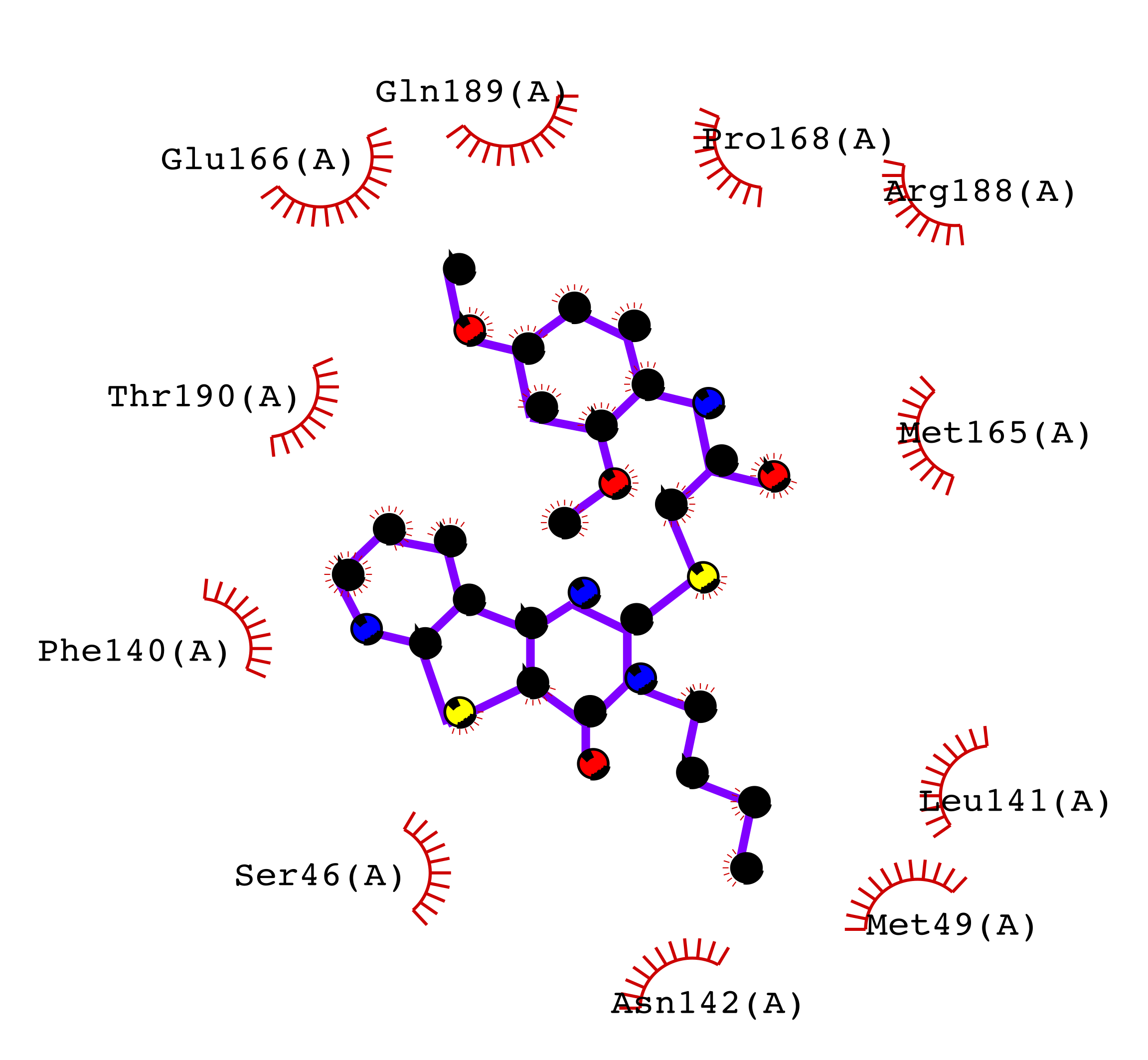 |
| IBS-E0474946 | 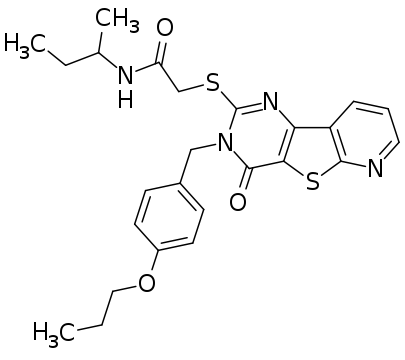 | -51.22 | 1.08 | 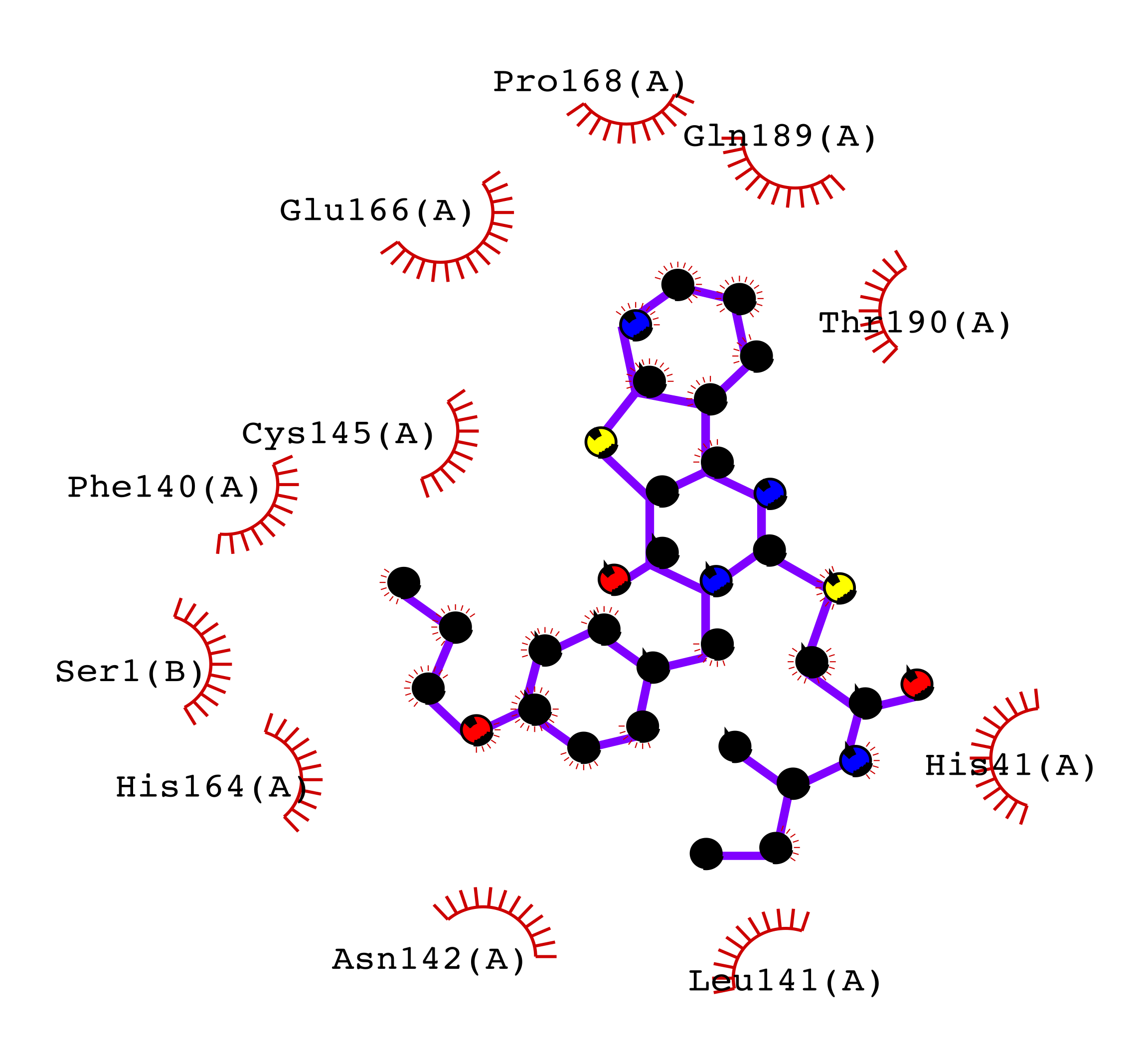 |
| IBS-E0475253 | 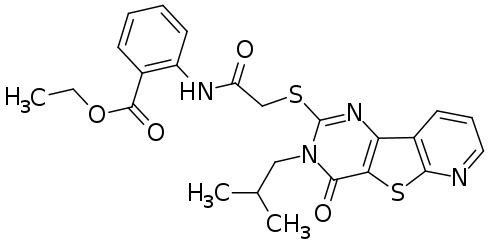 | -50.82 | 1.28 | 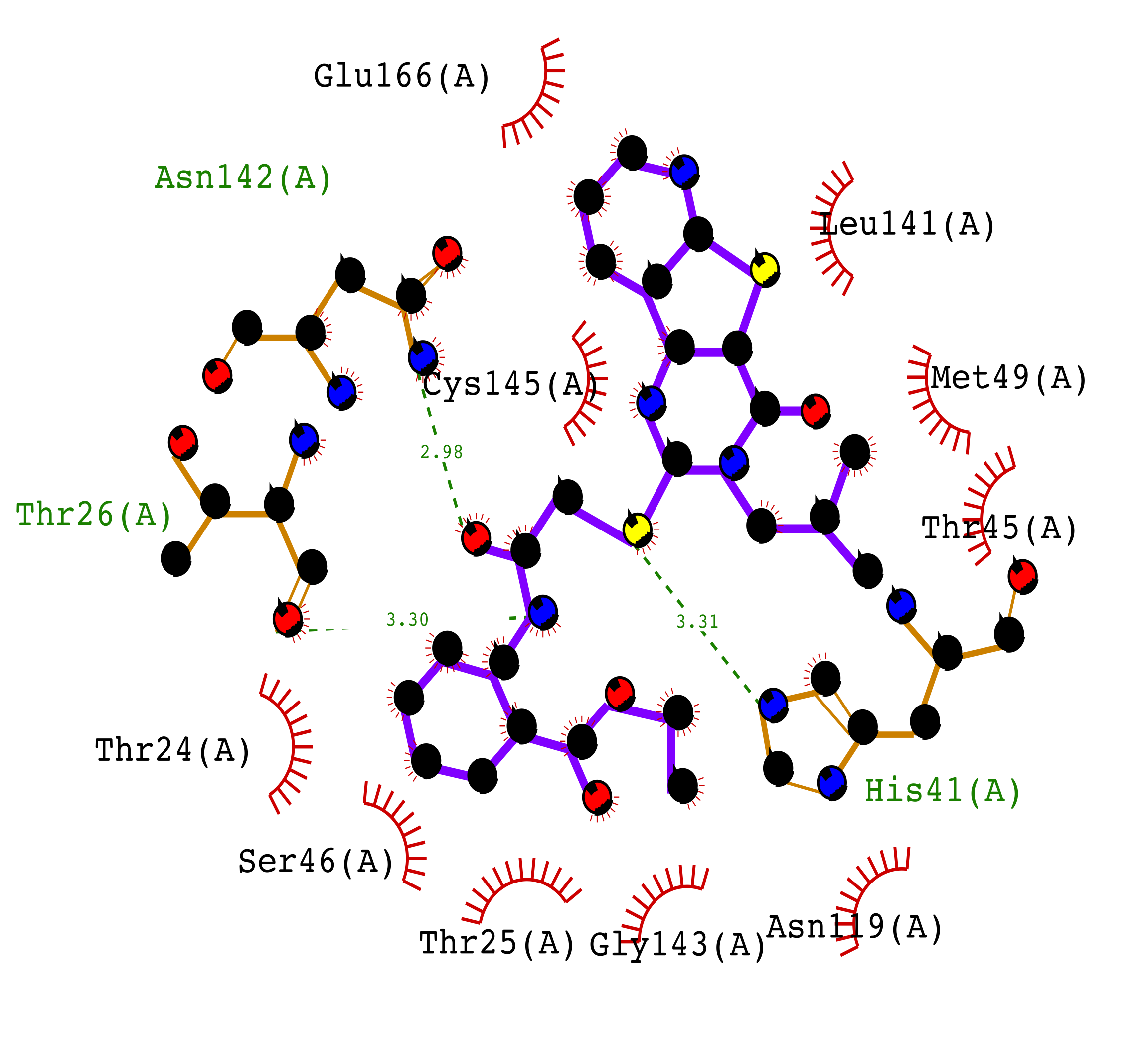 |
| IBS-E0530026 | 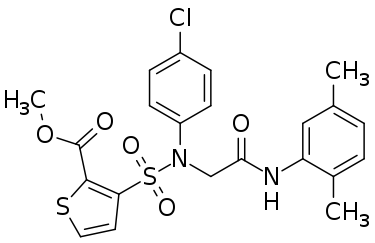 | -52.31 | 0.76 | 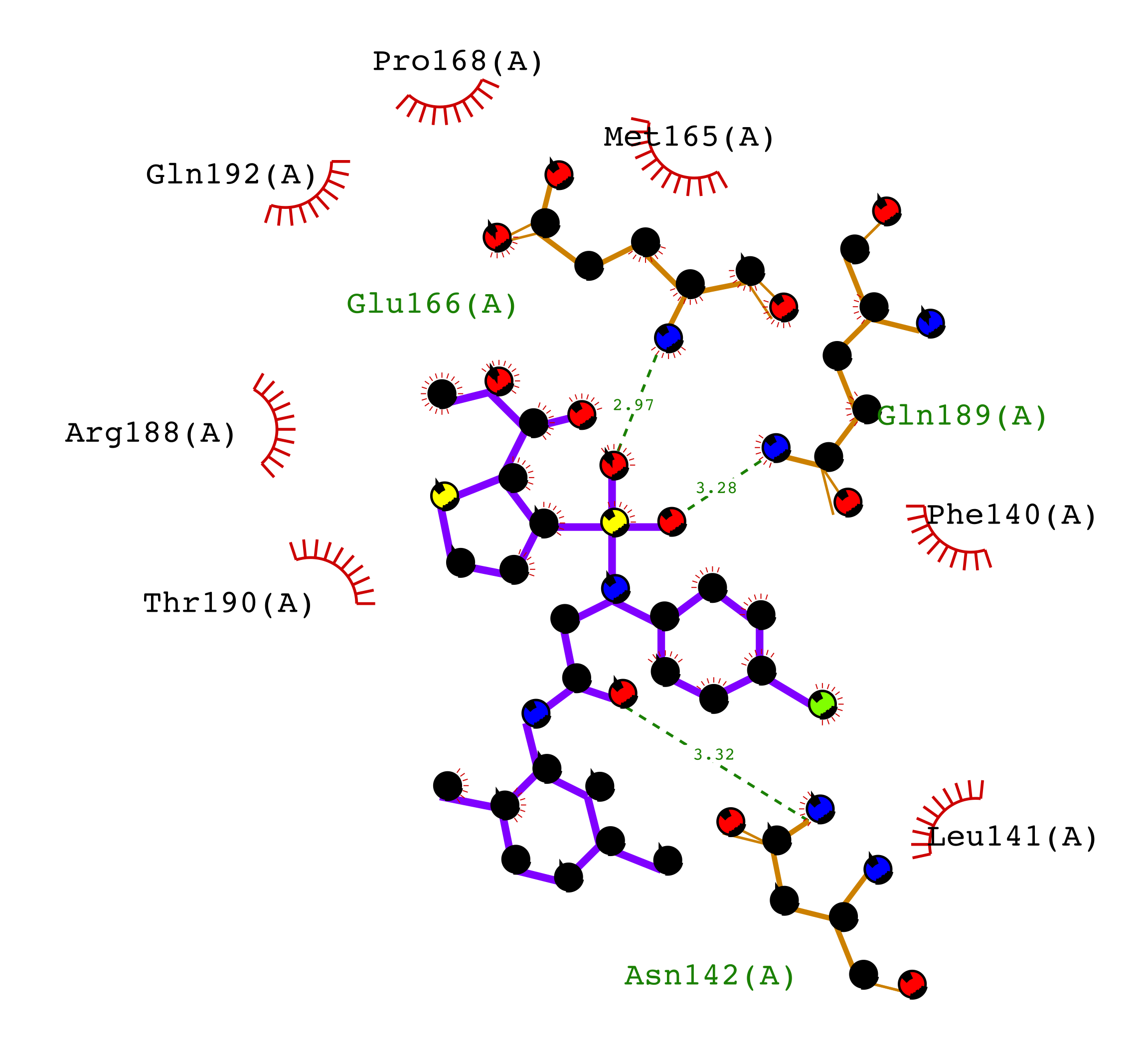 |
| IBS-E0529844 | 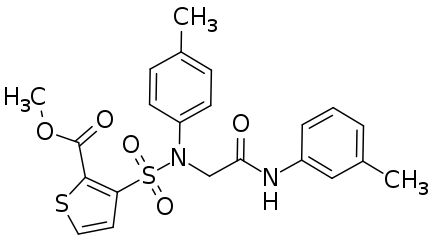 | -51.55 | 1.04 | 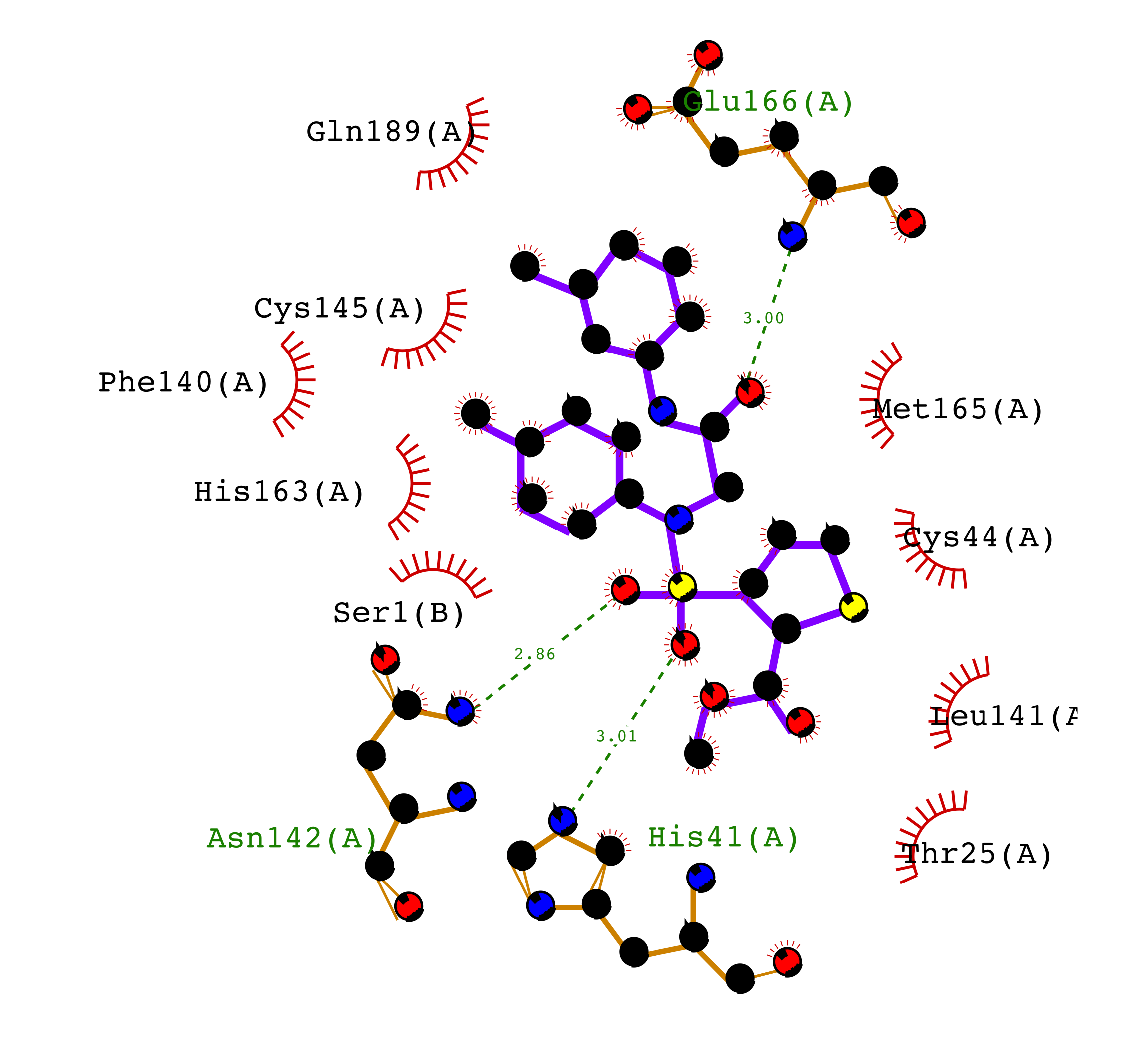 |
| **IBS-E0183442** | 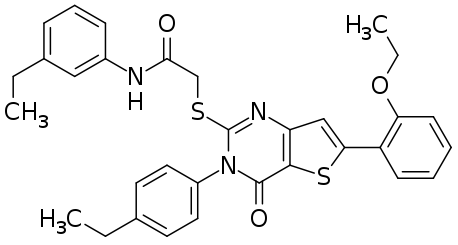 | -51.92 | 0.45 | 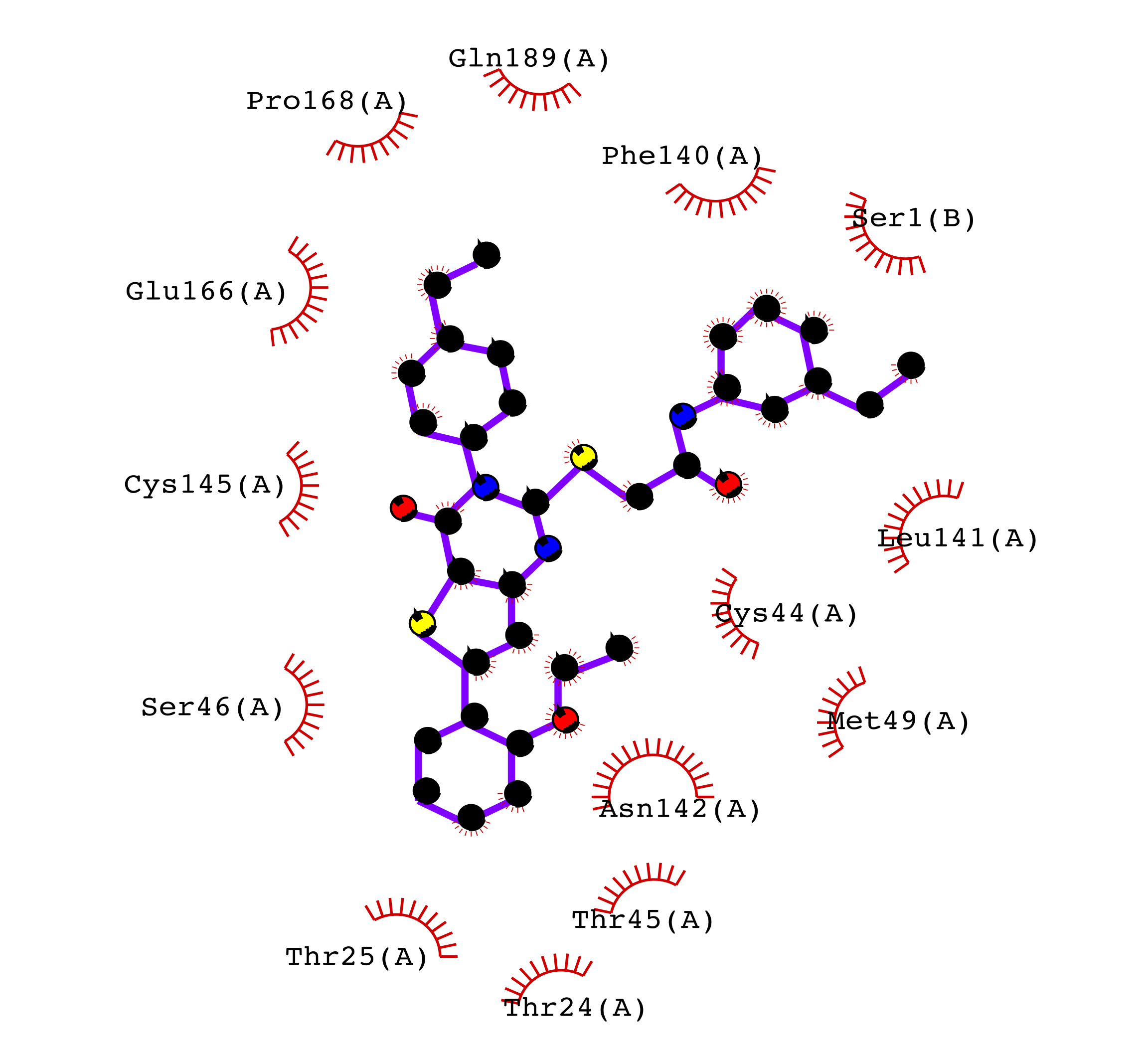 |
| **IBS-E0680092** | 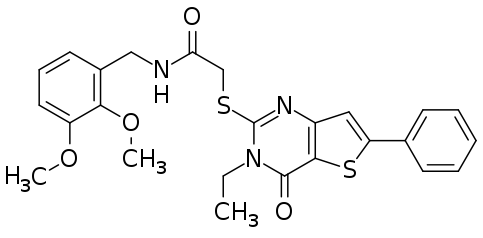 | -50.91 | 0.34 | 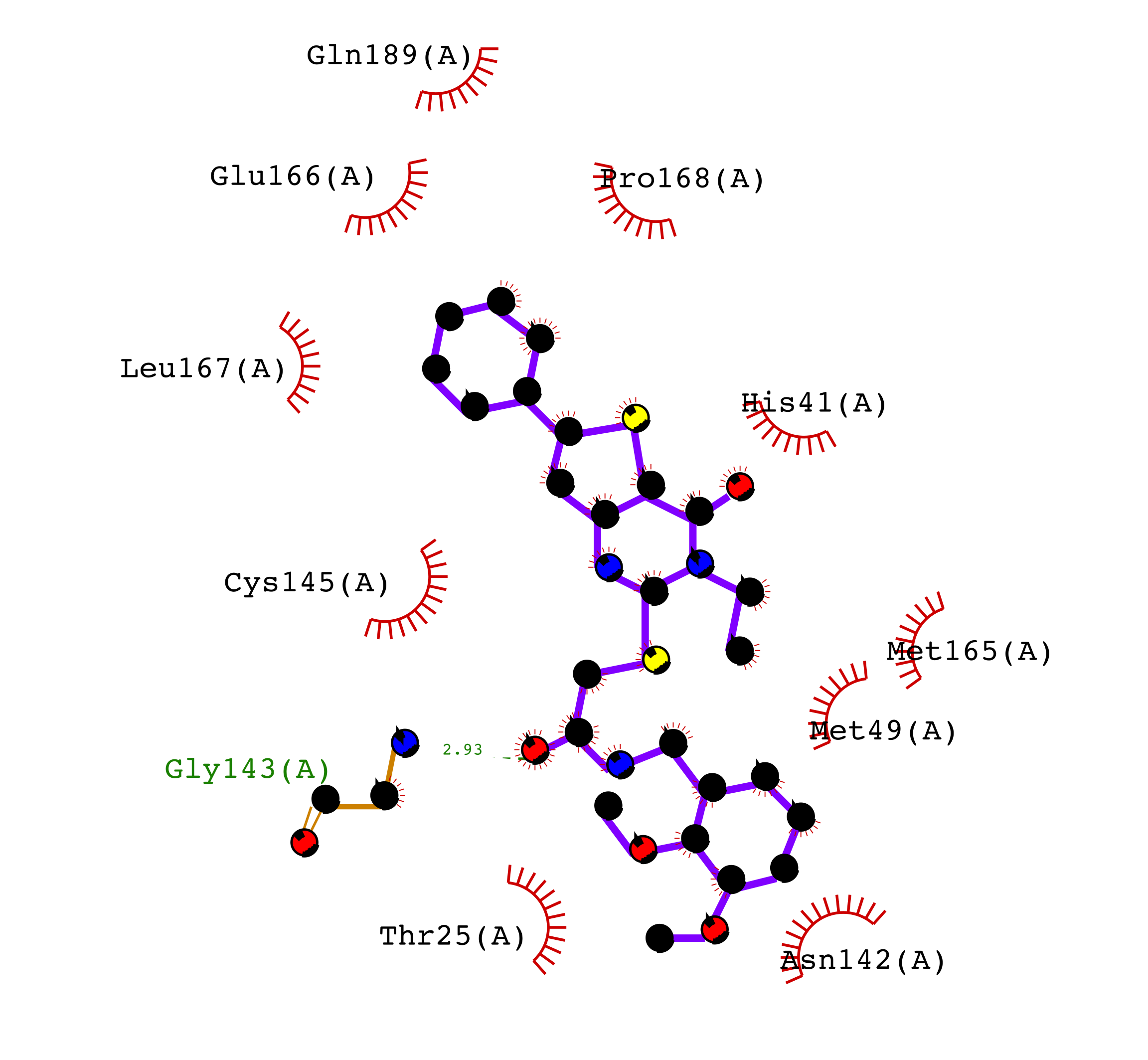 |
| IBS-E0148673 | 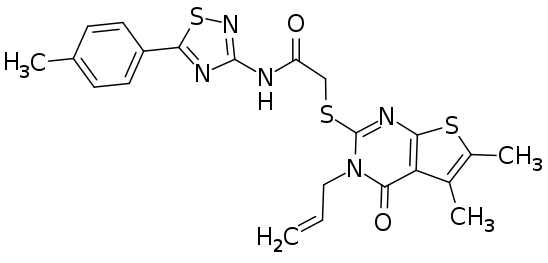 | -46.66 | 0.95 | 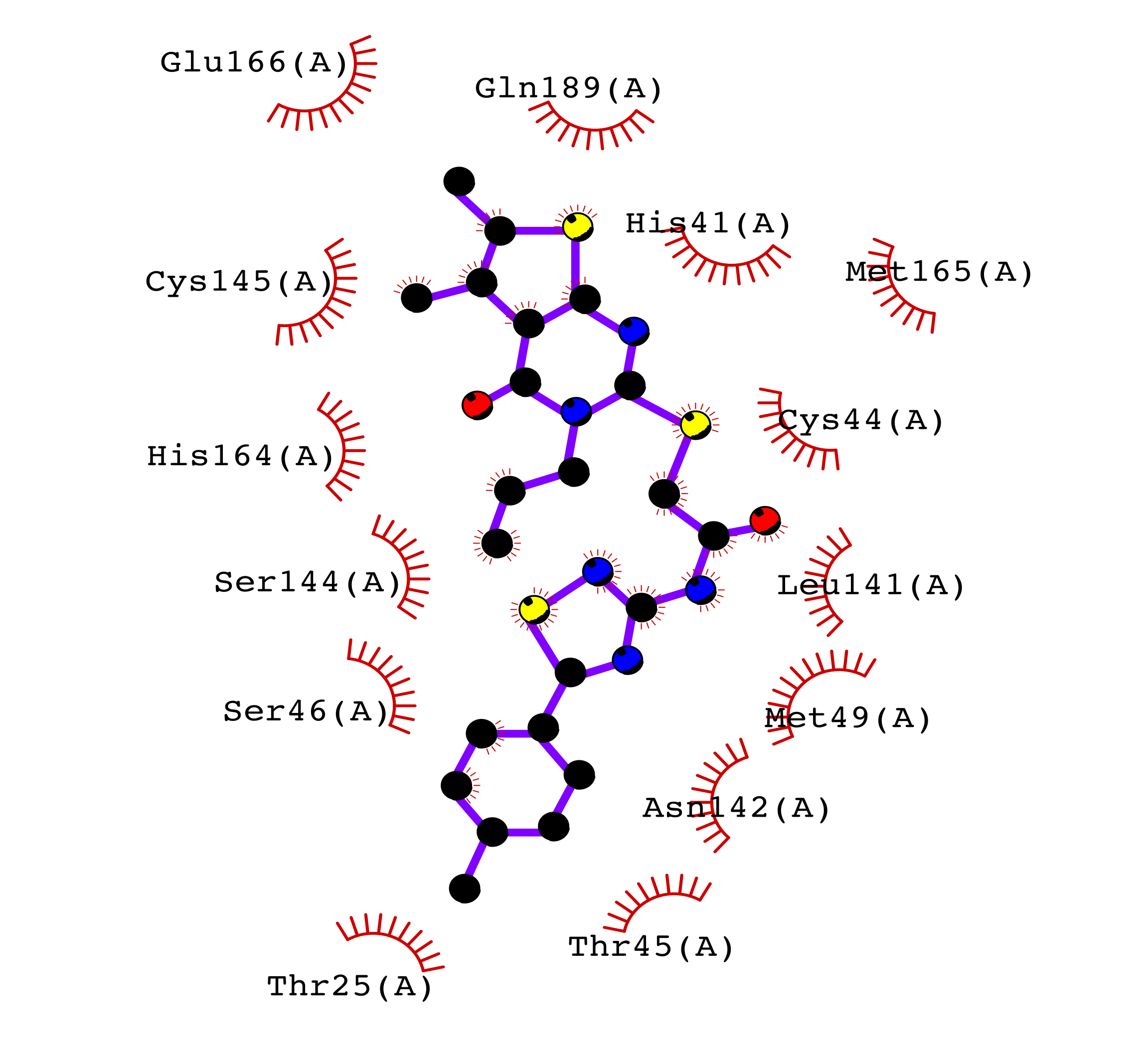 |





Figure S1. Dependence of the initial rate of substrate cleavage by SARS-CoV-2 Mpro fitted by Hill equation.
